# Optimising health and economic impacts of COVID-19 vaccine prioritisation strategies in the WHO European Region: a mathematical modelling study

**DOI:** 10.1016/j.lanepe.2021.100267

**Published:** 2021-11-30

**Authors:** Yang Liu, Frank G. Sandmann, Rosanna C. Barnard, Carl A.B. Pearson, Roberta Pastore, Richard Pebody, Stefan Flasche, Mark Jit

**Affiliations:** a Department of Infectious Disease Epidemiology, Faculty of Epidemiology and Population Health, London School of Hygiene and Tropical Medicine; Keppel St, London, United Kingdom WC1E 7HT; b Centre for Mathematical Modelling of Infectious Diseases, London School of Hygiene and Tropical Medicine; Keppel St, London, United Kingdom WC1E 7HT; c Statistics, Modelling and Economics Department, National Infection Service, Public Health England; 61 Colindale Ave, London, United Kingdom NW9 5EQ; d World Health Organization (WHO) Regional Office for Europe; UN City, Marmorvej 51, 2100, Copenhagen, Denmark

**Keywords:** Vaccine policy, Health economics, Policy evaluation, COVID-19, Europe, Mathematical modelling, Multicountry analysis

## Abstract

**Background:**

Countries in the World Health Organization (WHO) European Region differ in terms of the COVID-19 vaccine supply conditions. We evaluated the health and economic impact of different age-based vaccine prioritisation strategies across this demographically and socio-economically diverse region.

**Methods:**

We fitted age-specific compartmental models to the reported daily COVID-19 mortality in 2020 to inform the immunity level before vaccine roll-out. Models capture country-specific differences in population structures, contact patterns, epidemic history, life expectancy, and GDP per capita.

We examined four strategies that prioritise: all adults (V+), younger (20-59 year-olds) followed by older adults (60+) (V20), older followed by younger adults (V60), and the oldest adults (75+) (V75) followed by incrementally younger age groups. We explored four roll-out scenarios (R1-4) — the slowest scenario (R1) reached 30% coverage by December 2022 and the fastest (R4) 80% by December 2021. Five decision-making metrics were summarised over 2021-22: mortality, morbidity, and losses in comorbidity-adjusted life expectancy, comorbidity- and quality-adjusted life years, and human capital. Six vaccine profiles were tested — the highest performing vaccine has 95% efficacy against both infection and disease, and the lowest 50% against diseases and 0% against infection.

**Findings:**

Of the 20 decision-making metrics and roll-out scenario combinations, the same optimal strategy applied to all countries in only one combination; V60 was more or similarly desirable than V75 in 19 combinations. Of the 38 countries with fitted models, 11-37 countries had variable optimal strategies by decision-making metrics or roll-out scenarios. There are greater benefits in prioritising older adults when roll-out is slow and when vaccine profiles are less favourable.

**Interpretation:**

The optimal age-based vaccine prioritisation strategies were sensitive to country characteristics, decision-making metrics, and roll-out speeds. A prioritisation strategy involving more age-based stages (V75) does not necessarily lead to better health and economic outcomes than targeting broad age groups (V60). Countries expecting a slow vaccine roll-out may particularly benefit from prioritising older adults.

**Funding:**

World Health Organization, Bill and Melinda Gates Foundation, the Medical Research Council (United Kingdom), the National Institute of Health Research (United Kingdom), the European Commission, the Foreign, Commonwealth and Development Office (United Kingdom), Wellcome Trust

**Research in Context**

**Evidence before this study**

We searched PubMed and medRxiv for articles published in English from inception to 9 June 2021, with the search terms: (“COVID-19” OR “SARS-CoV-2”) AND (“priorit*) AND (“model*”) AND (“vaccin*”) and identified 66 studies on vaccine prioritization strategies. Of the 25 studies that compared two or more age-based prioritisation strategies, 12 found that targeting younger adults minimised infections while targeting older adults minimised mortality; an additional handful of studies found similar outcomes between different age-based prioritisation strategies where large outbreaks had already occurred. However, only two studies have explored age-based vaccine prioritisation using models calibrated to observed outbreaks in more than one country, and no study has explored the effectiveness of vaccine prioritisation strategies across settings with different population structures, contact patterns, and outbreak history.

**Added-value of this study**

We evaluated various age-based vaccine prioritisation strategies for 38 countries in the WHO European Region using various health and economic outcomes for decision-making, by parameterising models using observed outbreak history, known epidemiologic and vaccine characteristics, and a range of realistic vaccine roll-out scenarios. We showed that while targeting older adults was generally advantageous, broadly targeting everyone above 60 years might perform better than or comparably to a more detailed strategy that targeted the oldest age group above 75 years followed by those in the next younger five-year age band. Rapid vaccine roll-out has only been observed in a small number of countries. If vaccine coverage can reach 80% by the end of 2021, prioritising older adults may not be optimal in terms of health and economic impact. Lower vaccine efficacy was associated with greater relative benefits in prioritising older adults.

**Implication of all the available evidence**

COVID-19 vaccine prioritization strategies that require more precise targeting of individuals of a specific and narrow age range may not necessarily lead to better outcomes compared to strategies that prioritise populations across broader age ranges. In the WHO European Region, prioritising all adults equally or younger adults first will only optimise health and economic impact when roll-out is rapid, which may raise between-country equity issues given the global demand for COVID-19 vaccines.

Alt-text: Unlabelled box

## Introduction

The COVID-19 pandemic poses unprecedented challenges to public health, health systems and economies globally. While non-pharmaceutical interventions (NPIs, e.g. physical distancing) have effectively mitigated COVID-19 transmission,^1^ extraordinary effort and resources have also been committed to developing and rolling out COVID-19 vaccines.^2^ These global efforts have led to successful vaccine development at an unprecedented speed.

Some countries have signed bilateral advanced purchasing agreements with vaccine manufacturers to independently procure enough vaccine doses to cover significant proportions of their populations. Many low- and middle-income countries (LMICs) do not have the resources for such an option.^3^ Globally coordinated efforts to roll out COVID-19 vaccines are required to achieve equitable vaccine distribution and control the COVID-19 pandemic.

The global initiative “COVID-19 Vaccines Global Access” (COVAX) has been set up to ensure equitable vaccine access across countries ^4^. However, the speed at which vaccines become available through COVAX is constrained by production and logistical capacities. In the interim vaccine distribution forecast published in February 2021, COVAX was projected to deliver vaccines to cover approximately 3% of the total population in the 145 COVAX facility participant countries by mid-2021 and up to 20% of those populations by the end of 2021.^4^^,^^5^ For the World Health Organization (WHO) European Region specifically, 16 of 53 Members States may follow this projection as non-Advanced Market Commitment (AMC) donors.^4^

Several additional challenges remain while deciding on the optimal vaccine prioritisation strategies, besides the diverse supply conditions. First, although there is some evidence supporting prioritising older adults in COVID-19 vaccine roll-out,^6^^,^^7^ the specific approach has not been explored and could vary drastically.^8^ Second, evidence on vaccine prioritisation strategies has predominantly been based on models fitted to data from single or similar countries (predominantly non-LMICs). The generalisability of such evidence to different social contexts and epidemic history remains unclear. Third, public health decision-makers need to consider the trade-offs between public health outcomes. However, most existing evidence only presents mortality and infections as decision-making metrics.

To address these gaps in evidence, this work was commissioned by WHO/Europe to inform the European Technical Advisory Group of Experts on Immunization (ETAGE) group for the regionalisation of the WHO Strategic Advisory Group of Experts on immunization (SAGE) Roadmap for prioritising population groups for vaccines against COVID-19, based on the regional specific context and published preliminary recommendations of selected national immunization technical advisory groups (NITAG) in the Region.

More specifically, this study evaluates different age-based vaccine prioritisation strategies given different vaccine supply conditions between 2021 and 2022 in the WHO European Region. We aim to identify strategies that maximise the health and economic impacts of COVID-19 vaccines for each country, measured by five decision-making metrics ((1) mortality, (2) cases, (3) comorbidity-adjusted life expectancy (cLE) loss, (4) comorbidity- and quality-adjusted life-years loss (cQALY), and (5) human capital (HC) loss). These metrics allowed us to explore the trade-offs between minimising COVID-19 mortality and morbidity. We consider the known epidemiology of SARS-CoV-2 and explore how demographic factors, government COVID-19 response policy stringency, community mobility in each country, and a wide range of vaccine characteristics may affect the optimal vaccine allocation strategies in the WHO European Region. Stakeholders to whom our research is of great value and interest include vaccination and immunisation experts advising government implementations, country-level policymakers focusing on public health planning and health economics, and vaccine programme managers.

## Methods

### Model framework

**Fig. 1:**
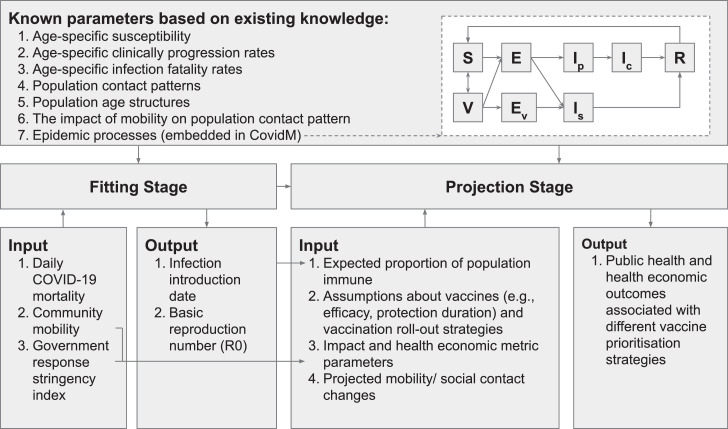
Model Framework This figure describes the overall model framework of the study, which consists of the fitting and projection stages. The “known parameters based on existing knowledge” were used in both fitting and projection stages. The remaining input was used in only one of the stages as specified.

Our model framework consisted of two stages (Fig. 1). The objective of the fitting stage was to estimate the proportion of each age group in every country who were no longer susceptible to SARS-CoV-2 by 01 January 2021. The projection stage relied on the results from the fitting stage, vaccine-related assumptions, health and economic impact parameters, and projected mobility changes related to expected public health and social measures. Using these, we estimated the health and economic impacts of different vaccine prioritisation strategies between January 2021 and December 2022 in each country.

For both stages, we used a previously described COVID-19 compartmental dynamic transmission model, CovidM.^9^ Equations describing the transmission dynamics can be found in the Supplemental Methods (p49-50). In brief, this model includes 16 age groups defined by 5-year age bands (from 0-4 to 75+) and eight compartments representing individuals who are: susceptible (S); exposed, i.e. infected but not yet infectious (E); vaccinated (V); vaccinated, exposed and progressing differently compared to unvaccinated and exposed individuals ($E_{v}$); infectious and showing no symptoms (i.e. pre-clinical) although symptoms would eventually appear ($I_{p}$); infectious and showing symptoms (i.e. clinical) ($I_{c}$); infectious and showing no symptoms during the course of infection (i.e. sub-clinical) ($I_{s}$); recovered from infection and protected by infection-induced immunity (R) (Fig. 1). The model assumes individuals in $I_{s}$ are 50% as infectious as those in $I_{p}$ and $I_{c}$; ^9^ age-specific clinical fractions (i.e. the proportion of exposed individuals (E) that eventually progresses through $I_{p}$) (range: 20⋅7%-69⋅1%);^10^ and age-specific susceptibilities.^10^ COVID-19 mortality is modelled as the product of age-specific infection counts (i.e. the sum of vaccinated and unvaccinated individuals progressing as E in Fig. 1, See Supplemental Methods p50) and the age-specific infection-fatality ratios (range: 6⋅7 per million infections – 8⋅1 per hundred infections, monotonically increasing by age).^11^

Models for each country were parameterised with country-specific age structures (2020, Fig. 2, A, Supplemental Figure 1),^12^ contact patterns (using synthetic contact matrices published in 2021 based on contact survey data from 2004-2018, Fig. 2, B),^13^ local NPIs stringency (January 2020-February 2021),^14^ community mobility (February 2020-February 2021),^15^ observed COVID-19 deaths (January 2020-December 2020),^16^ life expectancy (2019) ^17^ and GDP per capita (2018 or 2019).^18^ Pathogen related parameters (e.g. incubation period), vaccine profiles and characteristics (e.g. duration of vaccine-induced immunity) were assumed to be common across the entire region. A table summarising input variables and assumptions is presented in Supplemental Table 1.

**Fig. 2:**
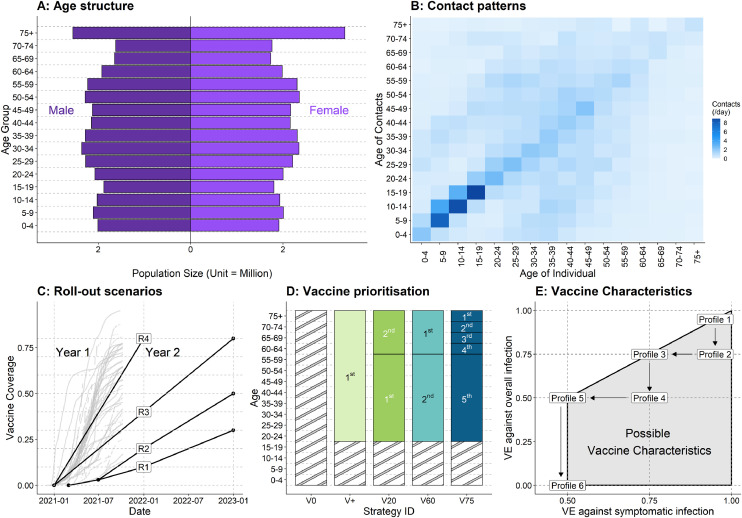
Key inputs and assumptions. (A) Example population age structure (for the United Kingdom, unit = million; those of other countries in the Region are presented in Supplementary Figure 1). [12] (B) Example age-specific within-population contact patterns (for the United Kingdom). [13] (C) Vaccine roll-out scenarios and the respective proportions of populations expected to be covered at different time points. Note that under different vaccine roll-out scenarios, the starting time of vaccination programs may differ. Grey lines in the background represent observed country-level vaccine uptakes (of the first dose) over time reported in the WHO European Region (as of October 2021). [25] (D) Vaccine prioritisation strategies. Hatched areas indicate when no vaccine was allocated. (E) Vaccine profiles consisting of vaccine efficacy against infection and disease.

### Fitting stage

Country-specific infection introduction dates and basic reproduction numbers (R_0_s) were obtained by deterministically fitting CovidM to daily reported COVID-19 mortality using maximum likelihood estimation (MLE) and differential evolution global optimisation algorithms assuming a Poisson likelihood function ^19^. The infection introduction date was constrained to a 90-day window before the first reported COVID-19 death in each country. The R_0_s were constrained to the 1.5 to 5 range.^20^ We generated 500 stochastic realisations of the outbreak trajectories over time to approximate the uncertainty needed to evaluate the overall performance of the fitting stage.

We used smoothed country-specific daily total reported COVID-19 mortality (i.e. right-aligned seven-day moving averages) in 2020 from *Our World in Data* for the WHO European Region, most of which has been collected from government reports.^16^ We fit the model to mortality rather than cases because the latter may suffer from greater underreporting due to limited testing capacity.^21^ The definition of a COVID-19 death in this dataset may vary slightly by country and over time and may not always require a positive COVID-19 test.^16^ In this study, we excluded countries with no (n = 1) or sparse mortality data (<10 deaths/ day throughout the study period, n = 12) or with major changes in the COVID-19 mortality case definition (n = 2).^22^ Of 53 members states of the WHO European Region, 38 were included in the fitting stage. The countries excluded and their corresponding rationales are listed in Supplemental Table 2.

Age-specific contact patterns (contacts per day by age-group and country) were captured by country-specific synthetic contact matrices, which include information from different contact settings: *work, school, home*, and *others*.^13^ However, social contacts have deviated from their pre-pandemic levels. Thus, we used Google community mobility data (expressed as percentage deviations from the pre-pandemic baseline established using data from January 2020) to approximate the changes in contact patterns in *work* and *other* settings during the pandemic.^15^^,^^23^ Google community mobility data is based on the movement history of users who have signed into their Google account, turned on their location history and used devices with Location Reporting turned on.^15^ The sample may not be representative of the entire population. Additionally, social contacts in the *school* setting were based on school-related NPI stringency from the OxCGRT database.^14^ This variable is originally categorical and ordinal, with values between 0 (no measure) and 3 (require closing all levels). This study assumes baseline *school* setting contacts at level 0, no *school*-based contacts at level 3, and 50% *school-*based contacts at level 1 or 2. Contacts at *home* were assumed to stay unchanged compared to pre-pandemic periods. More information on mobility and NPI stringency can be found in Supplemental Tables 3-5, Supplemental Figures 2-6.

Evidence suggests COVID-19 mortality may have been higher than reported in many countries ^24^. To account for this, we repeated the fitting stage estimating for an additional parameter — a country-specific temporally-invariant mortality underreporting rate, as a sensitivity analysis.

### Projection stage

We assume that contact patterns (*work* and *other*) gradually recover over a year after March 2021 but never fully return to pre-pandemic levels due to long-term policies and behavioural changes (Supplemental Tables 3-5, Supplemental Figures 2-6). More specifically, we assumed that NPI intensity would gradually decrease as vaccination is rolled out. We did not consider scenarios where NPIs may be reinstated if further outbreaks occur because the focus of our study was to evaluate vaccine strategies rather than NPIs. Contact patterns in the *school* setting followed school terms.^26^

We examined four possible roll-out scenarios: R1, R2, R3 and R4 (Fig. 2, C), based on supply projections, observed roll-out speed in different countries, and potential challenges to achieving high uptake. R2 resembles the projected roll-out projections under COVAX.^4^^,^^5^ In R1, vaccine roll-out is slower and final uptake lower compared to R2. Contributors to relatively slow roll-out speeds (such as in R1) may include vaccine production delay and logistical challenges in delivering vaccines. R1 and R2 both start from 01 March 2021.^27^ R4 resembles the trajectories of the rapid adopters of the region.^25^ R3 achieves the same coverage as R4 but at a slower pace. R3 and R4 both start from 01 January 2021.^25^ In the WHO European Region, as of August 2021, 25 countries have rolled out vaccination at a rate equal to or greater than R4, 14 countries at a rate between R3 and R4, nine countries at a rate between R1 and R3, and the remaining five countries have not yet reported vaccine uptake.^25^

We examined four vaccine prioritisation strategies (Fig. 2, D). In V+, the entire population aged 20+ years received vaccines simultaneously. In strategy V20, younger adults (20-59 year-olds) received vaccines before older adults (60+ year-olds). In both V60 and V75, older adults (60+ year-olds) were vaccinated first; however, in V60, they were all vaccinated simultaneously. In V75, age groups were prioritised in 5-year age bands, starting from those above 75 years of age.

The vaccine roll-out among age groups above 60 years was considered complete when uptake reached 90%, and among age groups between 20 and 59 years, it was considered complete when uptake reached 70%. These targets are consistent with optimistic roll-out objectives and intended vaccine uptake observed in the WHO European Region.^2^^,^^28^ We additionally explored conditions where vaccine uptake thresholds were lower (e.g. due to vaccine hesitancy; ^29^ “lower uptake targets” were 80% for those above 60 and 65% for 20-59 year-olds; “extremely low uptake targets” were 60% for those above 60 and 45% for 20-59 year-olds) (see Supplemental Methods, p53 for rationale) and when adolescents (10-19 year-olds) become eligible for the vaccines (while assuming same uptake targets as younger adults, see Supplemental Methods p54 for rationale).

There is still considerable uncertainty around the immunity against SARS-CoV-2. In this study, we assumed a 14-day delay between vaccination (i.e. receiving the vaccine) and immunisation (i.e. becoming protected by the vaccine).^30^ Based on a recent cohort study,^31^ we assumed that protection gained from natural SARS-CoV-2 infection would wane exponentially with a mean duration of 3 years. Evidence on the duration of vaccine-induced immunity has been limited. In this study, we conservatively assumed that vaccines would wane exponentially with a mean duration of 52 weeks, potentially due to factors such as vaccine escape. We additionally included a sensitivity analysis assuming a mean duration of vaccine-induced immunity of 3 years, consistent with that of natural immunity.^32^

Infection-blocking and disease-reducing mechanisms of vaccines were both modelled. As baseline vaccine efficacy (VE), we assume that vaccination prevents 95% of infections and diseases based on the highest performing vaccines in clinical trials and post-introduction observational data.^30^^,^^32^^,^^33^ Additionally, we explored a range of vaccine profiles (see Fig. 2, E) to investigate the impact of vaccine performance on optimal vaccine prioritisation strategies. Countries may receive a range of vaccine products,^32^ which in turn have different VE estimates depending on local SARS-CoV-2 variants. The highest performing vaccine (i.e. profile 1) resembles the Pfizer-BioNTech COVID-19 vaccine; profiles 3-4 resemble AstraZeneca's COVID-19 vaccine.^32^ The worst performing vaccine profile (profile 6) has 50% efficacy against diseases and does not protect against infection. Such a profile could correspond to the performance of some currently available vaccines against certain variants of concern (VOCs) with high vaccine escape potential. A sensitivity analysis was conducted incorporating an additional 50% increase in pathogen transmissibility since April 2021 to further account for possible VOC characteristics.^32^^,^^34^

We used an ordinal logistic regression model to explore whether the optimal vaccine prioritisation strategies identified are associated with country-specific characteristics such as population size, age structure, contact patterns, the proportions of non-susceptible individuals by 01 January 2021, and roll-out scenario. To reduce collinearity, when a pair of variables are strongly associated with each other (Pearson's correlation > 0.7), we only keep one variable of the pair that has smaller correlations with other variables considered (outside the pair).

### Outcomes

We assessed the impact of different vaccine prioritisation strategies in each country using the projected cumulative measure of five health and economic decision-making metrics summarised over 01 January 2021 — 31 December 2022: (1) mortality counts, (2) case counts, (3) comorbidity-adjusted life expectancy (cLE) loss, (4) comorbidity- and quality-adjusted life years (cQALY) loss, and (5) value of human capital (HC) loss. The optimal strategy minimises these outcomes. Since the top-ranking strategies may have similar effects, we additionally calculated the regional totals for each health and economic metric when one strategy is applied to the entire region. For direct health outcomes, we focused only on (1) mortality and (2) cases. The method used to calculate COVID-19 mortality is described above. Projected COVID-19 cases are calculated by summing all infected individuals progressing through $I_{c}$ in Fig. 1 (i.e. all symptomatic infections). We did not estimate for hospitalisations and intensive care unit (ICU) admission, as they rely on factors such as severity and healthcare system capacity, which vary by country and over time.^35^ We briefly define cLE, cQALY, and HC below and provide more details on their values in Supplemental Figures 7-9 and on their corresponding loss functions in Supplemental Methods p51-52.

Life expectancy is the number of years an individual would be expected to live had they not died prematurely.^17^ In the context of the COVID-19 pandemic, we calculate the cLE by assuming a 50% increase in the standardised mortality ratio (defined as how given comorbidity may increase the risk of dying).^36^ The cLE is country- and age-specific.

The cQALY is similar to the cLE but weigh the years someone is alive based on their health-related quality of life.^37^ The cQALY loss due to premature COVID-19 mortality was calculated using the average age-specific population norms available for seven European countries and country-specific life expectancies, assuming the QALY norms to be the same in other countries in the WHO European Region.^38^ We estimated that each symptomatic COVID-19 case leads to the loss of 0.0307 QALYs, which incorporates detriments due to non-hospitalised illness episodes, hospitalisations, intensive-care treatment, and “long COVID” (parameterised using UK data, see Supplemental Methods p51-52). Short-term vaccine side effects were assumed to occur at 50% probability and to lead to a loss of 1 quality-adjusted life day per vaccinee.^39^^,^^40^

The HC method weighs the number of life-years someone loses by the country-specific GDP per capita as a proxy for the value of that individual's lost lifetime productivity. These values are not adjusted for age or sex since there is growing consensus that doing so undervalues unpaid labour.^41^ We used the World Bank's country-level GDP per capita estimates.^42^

The analyses were conducted using R (4.0.4).^43^ An R Shiny application is available at https://cmmid-lshtm.shinyapps.io/demo/ to enable users to explore additional values for parameters such as roll-out speed, vaccination program starting date, and final up takes not explored in this study.

### Role of funding source

The funders of the study had no role in study design; data collection, analysis, and interpretation; preparation of the manuscript; or the decision to publish.

## Results

### Model fitting

**Fig. 3:**
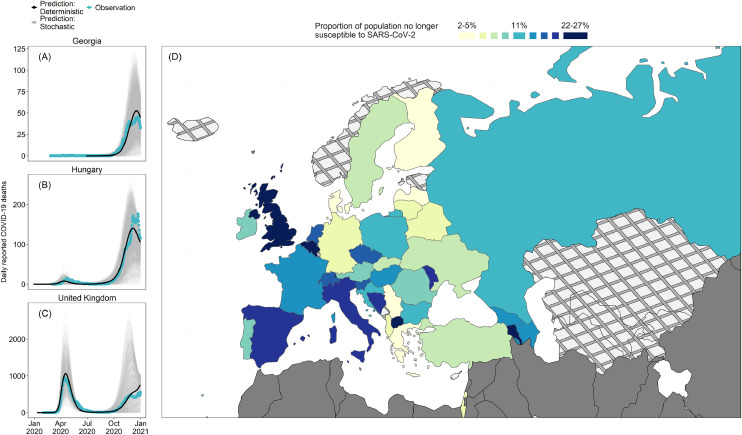
Results of the fitting stage (A-C) Comparisons between observed (blue line) and predicted COVID-19 deaths using a deterministic realisation based on fitted parameters (black line) and 500 stochastic outbreak realisations based on the same fitted parameters (grey lines) in Georgia, Hungary, and the United Kingdom. (D) The estimated proportions of individuals no longer susceptible (non-susceptible) to SARS-CoV-2 infection on 01 January 2021. Age-specific immunity level estimates were weighted by population age structure while calculating the country-level immunity levels. Countries marked by crosshatch patterns are those that were not included in the fitting stage; countries marked by the solid grey area outside the WHO European Region. Shapefiles were downloaded from Eurostat GISCO.[46]

The results of the fitting process for Georgia, Hungary and the United Kingdom are shown in Fig. 3, A-C. These were chosen as representatives of countries with daily COVID-19 mortality in tens, hundreds, to thousands, respectively. Results for all other countries are in our Zenodo archive.^44^

We found that the inferred infection introduction in most countries occurred in January 2020 (n = 38, IQR: 25 December 2019, 14 January 2020) (Supplemental Figure 10). The median R_0_ estimate was 1⋅67 (n = 38, IQR: 1⋅58, 1⋅85) (Supplemental Figure 11). These estimates are relatively low compared to existing literature,^45^ possibly due to NPIs that are not fully captured by the intensity of mobility and contact patterns (e.g. mask-wearing). Using these inferred parameters, we estimated the median proportion of individuals no longer susceptible to SARS-CoV-2 by 01 January 2021 to be 13⋅09% (n = 38, IQR: 6⋅94%, 21⋅63%) (Fig. 3, D).

### Optimal prioritisation strategies

We observed substantial differences in the optimal vaccine prioritisation strategy. Of 38 countries with fitted models, 7, 16, 27, and 22 countries have consistent optimal strategies across decision-making metrics within R1, R2, R3, and R4, respectively. Only one of the 38 countries with fitted models have the same optimal strategy across decision-making metrics and across roll-out scenarios. Of the 20 decision-making metric and roll-out scenario combinations (n = 20 = 5 metrics × 4 scenarios), only one identified the same optimal strategy across the entire region - while minimising morbidity under R1, V20 is the optimal strategy for all countries with fitted models.

**Fig. 4:**
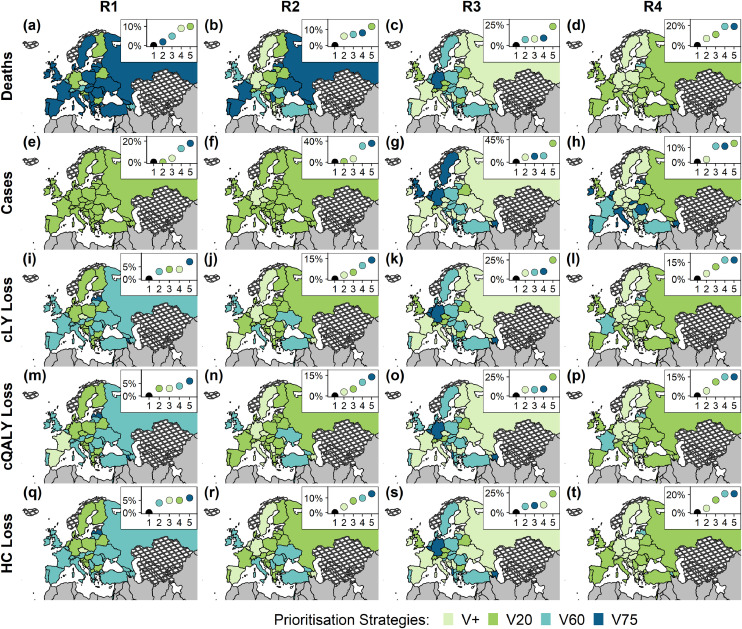
Optimal vaccine prioritisation strategies under different roll-out scenarios and decision-making metrics Main panel — Optimal strategies across the WHO European Region that minimise COVID-19 deaths, cases, losses in comorbidity-adjusted life expectancy (cLE), comorbidity- and quality-adjusted life-years (cQALY), and human capital (HC) as decision-making metrics. All decision-making metrics were summed over 01 January 2021-31 December 2022. Top right inserts within each panel — y-axis: Difference in outcome (totalled over the region) when a given prioritisation strategy is used across the entire WHO European Region compared to if the optimal prioritisation strategy in each country is used (black) x-axis: ranking. Shapefiles are downloaded from Eurostat GISCO [46]; countries marked by crosshatch patterns are those that were not included in the projection stage; countries marked by solid grey are those outside the WHO European Region. Country-specific results can be found in the Zenodo archive [44]

Under R1 and R2, in most countries, the optimal strategy is V75 to minimise COVID-19 deaths and V20 to minimise COVID-19 cases (Fig. 4 (a-b), (e-f)). Under R1 and based on cLE, cQALY or HC losses, most countries had V60 as their optimal strategy, although overall the performances all strategies were similar when applied to the entire region, leading to 3-7% (range, n = 12 = 4 strategies × 3 metrics) more losses compared to when local optimal strategies were used (Fig. 4, (i), (m), (g)). Under R2, although there were a comparable number of countries with V+, V20 and V60 as their optimal strategy based on cLE, cQALY and HC losses, V+ was the best performing strategy when applied to the entire region (Fig. 4, (j), (n), (r)).

Under R3, V+, V60 and V75 have roughly equal performance when applied to the entire region regardless of metric (Fig. 4, (c), (g), (k), (o), (s)), leading to an 8-13% (range, n = 15, 3 strategies × 5 metrics) increase compared to when the optimal strategy for each country was used instead using all metrics. However, under R4, not prioritising by age (V+) becomes the optimal strategy in most countries (Fig. 4, (d), (h), (l), (p), (t)). Proportions of countries and populations by optimal strategies, roll-out scenarios, and decision-making metrics are presented in Supplemental Tables 6-7. Overall, V60 performs better or comparably to V75 in 19 of 20 decision-making metrics and roll-out scenario combinations.

The results of the ordinal logistic regression model show that the only factors that were significantly associated (with type I error rate < 0.05) with the optimal vaccine prioritisation strategies were roll-out scenarios (Supplemental Figure 12). Faster roll-out scenarios were associated with having optimal strategies that target younger or all adults. Additionally, there was weak evidence (with type I error rate < 0.1) supporting the association between the intensity of adult to older adult contact patterns with having optimal strategies that prioritise older adults.

When the duration of vaccine-induced immunity is longer than expected (i.e. three years), the advantage of prioritising older adults tends to diminish (Supplemental Figure 13). A more transmissible VOC would make the advantages of V60 and V75 under R1-R3 and of V20 under R4 even more universal within the WHO European Region (Supplemental Figure 14). After incorporating underreporting of COVID-19 mortality, we estimated higher proportions of non-susceptible individuals than baseline scenarios. However, the optimal vaccine allocation strategies for the entire region remained broadly unchanged (Supplemental Figure 15, inner panels).

The optimal prioritisation strategies could be sensitive to the decision time frame (Supplemental Figure 16). The time frame in the baseline analysis (i.e. with a time horizon of December 2022) was decided following discussions with stakeholders. Such a long time horizon was agreed on because a shorter horizon would bias decisions against those that may prevent outbreaks that occur later in 2022. However, a time horizon beyond 2022 was decided against because of the large uncertainty around viral evolution and changes in human behaviour.

In our sensitivity analyses using lower vaccine uptake thresholds, we showed that the optimal prioritisation strategies stayed broadly unchanged (87% unchanged using “lower uptake targets”, and 61% unchanged using “extremely low uptake targets”, see Supplemental Figure 17-18). Lastly, our model shows that vaccinating adolescents with younger adults may bring additional health and economic benefits in 5-18 of 38 countries while using V60 and 25-28 of 38 countries while using V75 (Supplemental Table 8).

### Sensitivity analysis by vaccine characteristics

**Fig. 5:**
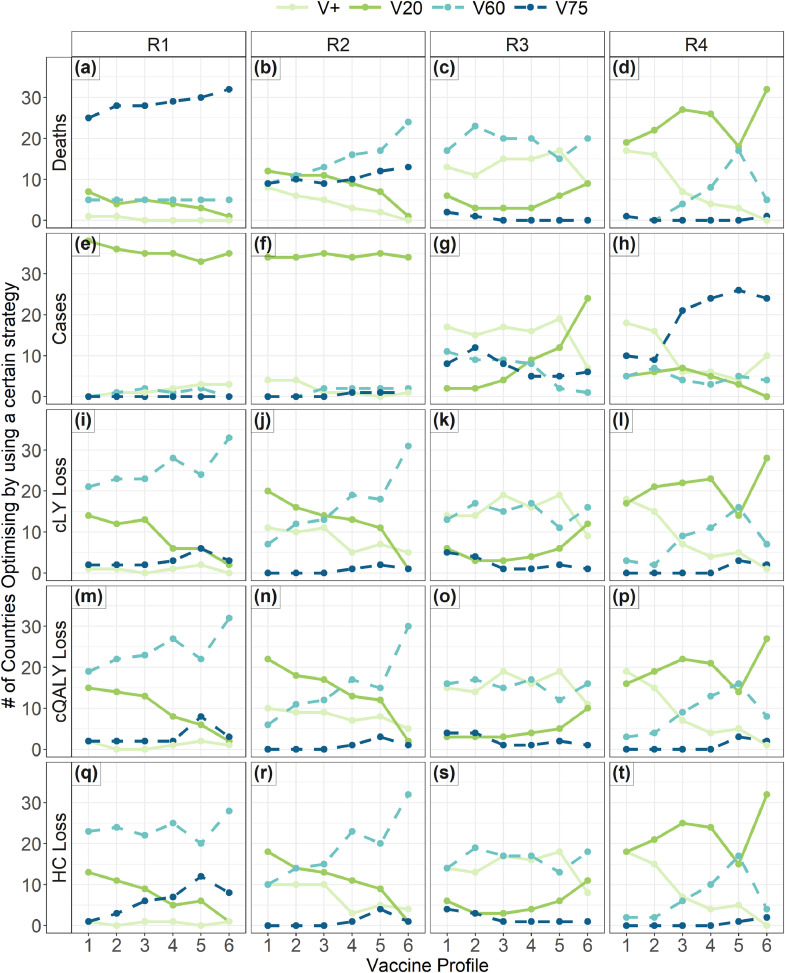
Optimal vaccine prioritisation strategies, given different vaccine profiles Optimal strategy for each country and vaccine profile while minimising mortality, morbidity, and losses in comorbidity-adjusted life expectancy (cLE), comorbidity- and quality-adjusted life-years (cQALY), and human capital (HC) for 38 countries in the WHO European Region. All decision-making metrics have been summed over 01 January 2021-31 December 2022. Country-specific results are in Supplemental Figures 19 and 20.

The optimal vaccine prioritisation strategy is sensitive to the vaccine profile (Fig. 5). Under R1 and R2, using low performing vaccines (i.e. profiles 5 and 6) is associated with having optimal strategies that prioritise older adults (i.e. V60 or V75) in terms of minimising deaths and losses in cLE, cQALY and HC. Under R3, the numbers of countries optimised by using V60 or V75 are relatively insensitive vaccine profiles across decision-making metrics. Under R4, low performing vaccines make V60 and V75 slightly more favourable than before. Under both R3 and R4, lower vaccine performances are both associated with more countries optimised by using V20 (except for Fig. 5, (h)) and less using V+. Stakeholders interested in country-specific results should refer to Supplemental Figures 19-20.

## Discussion

Using a transmission model of SARS-CoV-2 fitted to reported COVID-19 mortality data in the WHO European Region, this study evaluates different vaccine prioritisation strategies under a range of possible vaccine roll-out scenarios in a wide range of social and demographic contexts. We found that country characteristics affect optimal vaccine prioritisation strategies. We also found that age-based vaccine prioritisation strategies with more stages do not necessarily perform better. A two-stage approach that prioritises all older adults (V60) consistently performs better than or comparably to a five-stage approach that initially prioritises the oldest age group and only extends to the next five-year age group younger when vaccination goals of the last group have been met (V75). The choice between V60 and V75 may not depend on the health and economic impact but on context-dependent factors such as delivery feasibility.

When vaccine supply and delivery capacity are in place to reach 80% coverage by the end of 2021, the benefits of precisely targeting older adults diminish. Prioritising older adults may be more valuable to countries that expect a longer timeline for vaccine uptake to increase (e.g. nearly half of the WHO European Region Member States). These conclusions continue to hold when in the scenario with a 50% more transmissible VOC. These findings raise issues around within-country and between-country health equity. Countries with sufficient supplies of vaccines may find that they maximise benefits domestically by offering vaccination without any age restrictions, as this could expedite roll-out. However, there are insufficient supplies of COVID-19 vaccines globally to allow all countries to pursue such a strategy. Increased supplies for one country come at the expense of another.^47^

Evidence on infection-blocking and disease-reducing VE of different vaccine products is emerging. Here, we examined six different vaccine profiles, ranging from a highly effective vaccine that prevents both infection and disease at 95% to a vaccine that only prevents 50% of disease and does not provide any protection against infection. This range is designed around the vaccine products currently licensed in the WHO European Region. If vaccines become disease-reducing but not infection-blocking, more countries expecting slower roll-out may prioritise older adults (V60 or V75), given that the severity of disease burden increases by age.

### Strengths and limitations

First, while existing literature focuses on evaluating COVID-19 vaccine prioritisation in any city or country, our study is the first to investigate the impact of such strategies under different circumstances across a large region. Through working with country-specific characteristics (e.g. age structure, contact pattern, epidemic history, roll-out speed), we have highlighted the value of local evidence. Different age structures, contact patterns, and sizes of existing epidemics may affect the health and economic impacts of vaccination prioritisation strategies.

Second, we built our analyses around realistic vaccine roll-out scenarios based on projecting the broad patterns in vaccine roll-out rates across the region. Third, we evaluated a wide range of age-prioritisation strategies, not only under different roll-out scenarios but also at different supply levels (e.g. 3%, 20%, 50% or 80% coverage). The results inform policy recommendations, providing the necessary details for continuous implementation. The models and analyses presented here have benefited from continuous input since late 2020 from the WHO Regional Office for Europe and technical advisors from many countries in the region. Fourth, we included additional decision-making metrics beyond mortality and morbidity, providing a more comprehensive assessment of the various trade-offs that need to be considered between outcomes.^48^ Fifth, we considered various vaccine profiles to account for uncertainty around vaccine characteristics. Finally, new evidence about vaccine action, changes in vaccine supply constraints and the emergence of additional variants of concern (VOCs) may necessitate re-evaluation. To facilitate this, we have developed an online tool to help inform such policy decisions (available since the end of 2020).

We did not examine exposure risk- (e.g. highly connected individuals) or occupation-based (e.g. healthcare providers) vaccine prioritisation strategies. The evaluation of these strategies requires parameters governing their transmission risk (e.g. contact matrices by occupation, coverage, and effectiveness of personal protective equipment), most of which are not currently available. We did not project healthcare system outcomes such as hospital bed and ICU occupancy as there are substantial variations across countries and subnational regions and overtime in patient pathways, healthcare capacity and hospital organisation.^35^

The fitting stage is based on mortality data before 2021, i.e. before the start of vaccine roll-out and the large-scale emergence of VOCs that are more transmissible, more clinically severe, and potentially immune escaping.^32^ To inform the level of population immunity present before vaccination programmes started, we fit models qualitatively to time series of death counts for each country to reproduce the course of epidemics in 2020, hence approximately capturing immunity levels when vaccination programs in the WHO European Region started in early 2021. Work to extend the fitting window into 2021 would be useful to address future policy questions. However, it would require vaccine product-, age- and dose-specific uptake levels, country-specific underreporting rates for cases as well as for deaths (to address changes in infection fatality risks due to vaccination and VOCs), and VOC-specific surveillance data (adjust for testing intensity and account for reporting bias), and updated mobility-to-contacts relationships. The fitting stage includes a country-specific temporally invariant underreporting rate of COVID-19 mortality as a part of the sensitivity analysis. This rate may be time-varying.^49^ However, incorporating time dependencies would likely make parameter identification difficult.

The projection stage uses constant vaccine efficacy values before December 2022, which may not remain valid as VOCs spread, although we used a range of vaccine profiles that likely cover performance against the most common variants in the WHO European Region as of mid-2021.^50^ We modelled the vaccine in use as a single-dose product with a 14-day delay between vaccination and effective protection. These assumptions may be replaced by new evidence on VE and the immunity development process as they emerge. Finally, we assumed NPIs gradually return to near pre-pandemic levels as vaccines roll out between March 2021 and March 2022. We have not mechanistically captured reactive rule based NPIs, such as workplace closure when incidence exceeds a certain threshold, because these rules differ widely across countries and over time.

In this study, we provide an important piece of evidence to inform the decision-making processes for designing age-based vaccine prioritisation strategies. In practice, stakeholders involved in such processes may need to consider additional pieces of evidence, such as vaccine supply chain capacity and healthcare system structure and capacity. From early 2021, Israel and Portugal have adopted V60-type approaches, drawing lines at 60 and 50 years respectively while prioritising older adults. The United Kingdom and Germany have adopted the V75-type approach, starting from individuals above 80 years old.^8^ Some European countries have chosen not to prioritise by age at all.^8^

### Conclusion

In conclusion, we identified optimal age-based vaccine prioritisation strategies under different vaccine roll-out scenarios and vaccine effectiveness profiles at the country level in the WHO European Region. We showed that the benefits of prioritising older adults were more evident for relatively slow vaccine roll-out scenarios. When prioritising older adults, broadly targeting everyone above 60 years consistently performed comparably or better than targeting the oldest adults first followed by the next younger five-year age group - the additional stages does not lead to health or economic gains. Prioritising younger adults or not prioritising by age is only beneficial when vaccines can be rolled out quickly (e.g. reaching 80% of the population vaccinated in 1 year).

## Contributors

YL, FS, MJ, and RPebody conceptualised research ideas; YL and MJ conducted literature search and synthesised existing evidence; YL and FS curated data; YL, FS, RCB, CABP were involved in data analyses and software/ application development; YL wrote the original draft; all named co-authors are involved in results visualisation, interpretation, and writing - review & editing; working group members conducted pre-submission peer-review.

## Data Availability Statement

We used publicly available data in this study, cited in the reference list or in the Supplemental Material. The CovidM modelling framework used has been published previously and is available on the CMMID COVID-19 GitHub page. All code used and country-specific intermediate results have been archived via Zenodo. An R Shiny application is available at https://cmmid-lshtm.shinyapps.io/demo/ to enable users to explore additional sets of parameters not covered in this study. A TREND statement checklist is presented in Supplemental Table 9.

Editor note: The Lancet Group takes a neutral position with respect to territorial claims in published maps and institutional affiliations.

## Declaration of Competing Interest

YL and MJ report grants from the National Institute of Health Research outside the submitted work (16/137/109). RCB and MJ are participants of the Scientific Pandemic Influenza Group on Modelling. The views expressed in this publication are those of the author(s) and not necessarily those of the European Commission, National Institute of Health Research (NIHR) (UK), Public Health England (PHE) or the Department of Health and Social Care (UK). The authors alone are responsible for the views expressed in this publication, and they do not necessarily represent the decisions or policies of the World Health Organization.

## Appendix Supplementary materials

Image, application 1
