# Supplementary material for: Optimising health and economic impacts of COVID-19 vaccine prioritisation strategies in the WHO European Region: a mathematical modelling study
Source: Lancet Reg Health Eur. 2021 Nov 30;12:100267. doi: 10.1016/j.lanepe.2021.100267 (PMC8629724; doi:10.1016/j.lanepe.2021.100267)
Supplement: Supplementary file 1 [file mmc1.docx]

**Supplemental Material**

Optimising health and economic impacts of COVID-19 vaccine prioritisation strategies in the WHO European Region: a mathematical modelling study

Yang Liu PhD^1, 2*^, Frank G. Sandmann PhD^1, 2, 3^, Rosanna C. Barnard PhD^1, 2^, Carl A.B. Pearson PhD^1, 2^, CMMID COVID-19 Working Group, Roberta Pastore MD^4^, Richard Pebody PhD^4^, Stefan Flasche PhD^1, 2^, Mark Jit PhD^1, 2^

1 Department of Infectious Disease Epidemiology, Faculty of Epidemiology and Population Health, London School of Hygiene and Tropical Medicine; Keppel St, London, United Kingdom WC1E 7HT

2 Centre for Mathematical Modelling of Infectious Diseases, London School of Hygiene and Tropical Medicine; Keppel St, London, United Kingdom WC1E 7HT

3 Statistics, Modelling and Economics Department, National Infection Service, Public Health England; 61 Colindale Ave, London, United Kingdom NW9 5EQ

4 World Health Organization (WHO) Regional Office for Europe; UN City, Marmorvej 51, 2100, Copenhagen, Denmark

* Corresponding author: [yang.liu@lshtm.ac.uk](mailto:yang.liu@lshtm.ac.uk); Keppel Street, London, WC1E 7HT, United Kingdom

**CMMID COVID-19 Working Group Members:**

Akira Endo, Gwenan M Knight, Joel Hellewell, Matthew Quaife, Oliver Brady, Rachael Pung, Yalda Jafari, Sam Abbott, Adam J Kucharski, Sebastian Funk, Rosalind M Eggo, W John Edmunds, Amy Gimma, Billy J Quilty, Samuel Clifford, James D Munday, Nikos I Bosse, Hamish P Gibbs, Nicholas G. Davies, Timothy W Russell, Christopher I Jarvis, Alicia Rosello, Kiesha Prem, Graham Medley, Simon R Procter, C Julian Villabona-Arenas, Damien C Tully, Katherine E. Atkins, Sophie R Meakin, Rachel Lowe, Kaja Abbas, Kathleen O'Reilly, Mihaly Koltai, William Waites, David Hodgson, Emilie Finch, Ciara V McCarthy, Paul Mee, Lloyd A C Chapman, Fiona Yueqian Sun, Stéphane Hué, Kerry LM Wong

Table of Contents

[1. Supplemental Tables 5](#_Toc86758353)

[**1.1 Parameter Table** 5](#_Toc86758354)

[**Table S1. Inputs and assumptions** 5](#_Toc86758355)

[**1.2 Country code reference table** 9](#_Toc86758356)

[**Table S2. Country names and their corresponding World Bank country code.** 10](#_Toc86758357)

[**1.3 Linking stringency indices to community mobility** 11](#_Toc86758358)

[**Table S3. Covariates table for the general additive model (GAM) used in mobility projection (from March 2021 to December 2022)** 11](#_Toc86758359)

[**1.4 Current country-specific stringency indices** 12](#_Toc86758360)

[**Table S4. Stringency indices on at the end of the observation window (i.e., 22 Feb 2021)** 12](#_Toc86758361)

[**1.5 Degree of missingness in mobility data and stringency index** 13](#_Toc86758362)

[**Table S5. Missingness in community mobility reports and stringency indices.** 13](#_Toc86758363)

[**1.6 Proportions of countries by optimal vaccine prioritization strategies** 14](#_Toc86758364)

[**Table S6. Proportions of countries with specific optimal vaccine prioritization strategies.** 15](#_Toc86758365)

[**1.7 Proportions of population by optimal vaccine prioritization strategies** 16](#_Toc86758366)

[**Table S7. Proportions of populations with specific optimal vaccine prioritization strategies.** 17](#_Toc86758367)

[**1.8 Vaccination strategies for adolescents** 18](#_Toc86758368)

[**Table S8. Counts of cases where a policy is ranked first in terms of optimising health and economic benefits (including ties)** 18](#_Toc86758369)

[**1.9 TREND (Transparent Reporting of Evaluations with Nonrandomized Designs) statement checklist** 19](#_Toc86758370)

[2. Supplemental Figures 25](#_Toc86758371)

[**2.1 Age pyramid in the WHO European Region** 25](#_Toc86758372)

[**Figure S1. Population age pyramid by country.** 25](#_Toc86758373)

[**2.2 Results projecting stringency index into the future** 28](#_Toc86758374)

[**Figure S2. Stringency indices by country after incorporating the assumption on mobility recovery.** 28](#_Toc86758375)

[**2.3. Projected population contacts – *work* setting** 29](#_Toc86758376)

[**Figure S3. Projected multipliers of daily contacts before December 2022 in the *work* setting** 29](#_Toc86758377)

[**2.4 Projected population contacts – *school* setting** 30](#_Toc86758378)

[**Figure S4. Projected multipliers of daily contacts before December 2022 in the *school* setting.** 30](#_Toc86758379)

[**2.5 Projected population contacts – *others* setting** 31](#_Toc86758380)

[**Figure S5. Projected multipliers of daily contacts before December 2022 in the *others* setting.** 31](#_Toc86758381)

[**2.6 Projected population contacts – *home* setting** 32](#_Toc86758382)

[**Figure S6. Projected multipliers of daily contacts before December 2022 in the *home* setting.** 32](#_Toc86758383)

[**2.7 Numeric comparison between adjLE, LEdisc, and adjQALEdisc** 33](#_Toc86758384)

[**Figure S7. Numeric comparison between crude life expectancy and comorbidity adjusted life expectancy (adjLE), discounted life expectancy (LEdisc) and discounted comorbidity- and quality-adjusted life expectancy (adjQALEdisc).** 33](#_Toc86758385)

[**2.8 Values of Comorbidity adjusted life years** 34](#_Toc86758386)

[**Figure S8. Comorbidity adjusted life expectancy by age and by country** 34](#_Toc86758387)

[**2.9 Values of GDP per capita** 35](#_Toc86758388)

[**Figure S9. Gross Domestic Production per capita (GDPpc, log-scaled) in the WHO European Region** 35](#_Toc86758389)

[**2.10** **[Two-variable fitting process] Estimated Infection Introduction Dates** 36](#_Toc86758390)

[**Figure S10. Fitted infection introduction dates in the WHO European Region.** 36](#_Toc86758391)

[**2.11** **[Two-variable fitting process] Estimated Infection Introduction Dates** 37](#_Toc86758392)

[**Figure S11. Fitted basic reproduction numbers in the WHO European Region.** 37](#_Toc86758393)

[**2.12 Results of ordinal logistic regression exercise** 38](#_Toc86758394)

[**Figure S12. Coefficients and their corresponding 90% and 95% confidence interval in the ordinal logistic regression model.**  38](#_Toc86758395)

[**2.13 [Sensitivity analysis] Longer waning period for vaccine-induced immunity** 39](#_Toc86758396)

[**2.14 [Sensitivity Analysis] VOC transmissibility adjustment** 40](#_Toc86758397)

[**Figure S14. Sensitivity analyses results showing changes in optimal vaccine prioritisation strategies using a pathogen that becomes 50% more transmissible on 15 April 2021.** 40](#_Toc86758398)

[**2.15 [Sensitivity analysis] Underreporting** 41](#_Toc86758399)

[**2.16 [Sensitivity analysis] Different decision time frames** 42](#_Toc86758400)

[**Figure S16. Optimal vaccine prioritisation strategies under different roll-out scenarios when decision-making metrics were summarised over different decision-making time frames.** 42](#_Toc86758401)

[**2.17 [Sensitivity Analysis] “Lower uptake targets”** 43](#_Toc86758402)

[**Figure S17. Sensitivity analyses results showing changes in optimal vaccine prioritisation strategies using the “current condition” set of uptake parameters.** 43](#_Toc86758403)

[**2.18 [Sensitivity Analysis] “Extremely low uptake targets”** 44](#_Toc86758404)

[**Table S18. Sensitivity analyses results showing changes in optimal vaccine prioritisation strategies using the “extremely low” set of uptake parameters.** 44](#_Toc86758405)

[**2.19 Country-specific vaccine-prioritisation strategies by different vaccine profiles under R2 and R3** 45](#_Toc86758406)

[**Figure S19. Optimal vaccine prioritisation strategies for different vaccine characteristics under R2 and R3.** 46](#_Toc86758407)

[**2.20 Country-specific vaccine-prioritisation strategies by different vaccine profiles under R1 and R4** 47](#_Toc86758408)

[**Figure S20. Optimal vaccine prioritisation strategies for different vaccine characteristics under R1 and R4.** 48](#_Toc86758409)

[3. Supplemental Methods 49](#_Toc86758410)

[**3.1 Algorithmic details of the transmission model** 49](#_Toc86758411)

[**3.1.1 Equations and Syntax** 49](#_Toc86758412)

[**3.2 Calculating COVID-19 mortality from the mathematical model** 50](#_Toc86758413)

[**3.3 Incorporating vaccine efficacy against disease observed into the transmission model** 50](#_Toc86758414)

[**3.2** **More details on health and economic impact metrics** 51](#_Toc86758415)

[**3.2.1 Comorbidity-adjusted life expectancy, comorbidity- and quality-adjusted life expectancy, and discounted life expectancy** 51](#_Toc86758416)

[**3.2.2 QALY associated with COVID-19 morbidity** 51](#_Toc86758417)

[**3.2.3 GDP per capita used in the human capital approach** 51](#_Toc86758418)

[**3.3 Impact and Health Economic Metrics** 52](#_Toc86758419)

[age-specific discounted life expectancy (LEdisc) * age-specific COVID-19 mortality * GDP per capita**3.4 [Sensitivity analysis] Vaccine Uptake** 52](#_Toc86758420)

[**3.5 Vaccinating adolescents** 54](#_Toc86758421)

[References 55](#_Toc86758422)

# **Supplemental Tables**

## **1.1 Parameter Table**

| Known Parameters based on Existing Knowledge | | | | |
| --- | --- | --- | --- | --- |
| Index | Variable | | Values | Source |
| 1 | Age-specific susceptibility ($u_{i}$) | | 0.38 - 0.88 | Davies et al.^1^ |
| 2 | Age-specific clinical progression rates ($y_{i}$) | | 0.21 - 0.70 | Davies et al.^1^ |
| 3 | Age-specific infection fatality rates | | Raw input: 5.2e-6 - 0.13  By age group: 6.7e-6 – 8.1e-2 | Levin et al.^2^ |
| 4 | Age- and country-specific within-population contact pattern ($C_{i,j,0}$) | | Country-specific | Prem et al.^3^ |
| 5 | Country-specific population age structures | | Country-specific | United Nations^4^ |
| 5 | Relationship between mobility and population contact pattern | | Defined by linear and nonlinear functions for the *workplace* and *other* settings, respectively. | Davies et al.^5^ by fitting to UK data. |
| 6 | CovidM | Latent period ($1/\sigma$) | ~gramma (μ = 2.5, k = 2.5) | Pearson et al.^6^  Davies et al.^5^  Davies et al.^7^  Bi et al.^8^  Liu et al.^9^  Linton et al.^10^  Nishiura et al.^11^ |
|  |  | Duration of preclinical infectiousness (${1/\gamma}_{p}$) | ~gramma (μ = 1.5, k = 4) |  |
|  |  | Duration of clinical infectiousness (${1/\gamma}_{c}$) | ~gramma (μ = 3.5, k = 4) |  |
|  |  | Duration of subclinical infectiousness (${1/\gamma}_{s}$) | ~gramma (μ = 5, k = 4) | Assumed, consistent with Davies et al.^7^ |
|  |  | Relative infectiousness of subclinical infections compared to clinical infections ($f$) | 0.5 | Assumed, consistent with Davies et al.^7^ |
|  |  | Mean duration of immunity from infection (1/$\omega_{n}$) | 3 years | Hall et al.^12^ |

### **Table S1. Inputs and assumptions**

**Caption:** The variable index numbers correspond to their numberings in Figure 1 of the main text.

| Fitting Stage - input | | |
| --- | --- | --- |
| Index | Variable | Source |
| 1 | Country-level daily COVID-19 Mortality (including 7-day rolling average) | Roser et al.^13^  Human Rights Watch^14^ on change in mortality case definition in Kyrgyzstan/ Kazakhstan |
| 2 | Observed country-specific community mobility | Google LLC.^15^ |
| 3 | COVID-19 Government Response Stringency Index and Government Response Tracker by country | Hale et al.^16^ |

**Table S1. Inputs and assumptions (Continued)**

| Projection Stage - input and assumptions | | | | |
| --- | --- | --- | --- | --- |
| Index | Variable | | Values | Source |
| 2 | Vaccine characteristics | Vaccine protection duration (1/$\omega_{v}$) | Baseline = 52 weeks  Sensitivity analysis:  3 years | Assumed |
|  |  | Infection blocking efficacy ($e_{i})$ | Baseline = 0.95, varied between 0 and 0.95 |  |
|  |  | Disease blocking efficacy | Baseline = 0.95, varied between 0.5 and 0.95 |  |
|  |  | Vaccine roll-out scenarios | 0.03 by mid-2021 and 0.2 by end of 2021, relatively slow roll-out may start after March 2021 | Gavi, the vaccine alliance^17^, World Health Organisation^18,19^ |
|  |  | Maximum willingness to receive vaccination | 0.7 for those between 20-59 and 0.9 for those above 90 | Wouter et al.^20^  Robinson et al.^21^  UK Government^22^ |
| 3 | Impact and health economic metrics | Country-specific comorbidity Adjusted Life Expectancy | Age-specific | p51-52 |
|  |  | Mean QALY associated with COVID-19 morbidity | 0.0307 | p51-52 |
|  |  | Median QALD associated with AEFI | 1 | Oliver et al.^23^ |
|  |  | Probability of adverse events following immunisation | 50% | Pfizer and BioNTech^24^ by summing roughly summing |
|  |  | Country-specific GDP per capita | Country specific | p51-52 |
| 4 | Projected mobility changes | |  | GAM Model estimated  Association between mobility and contact is based on Davies et al.^5^ |

**Table S1. Inputs and assumptions (Continued)**

## **1.2 Country code reference table**

| **Country Name** | **World Bank Country Code** | **Country Name** | **World Bank Country Code** |
| --- | --- | --- | --- |
| Albania | ALB | Lithuania | LTU |
| Andorra | AND^*^ | Luxembourg | LUX^*^ |
| Armenia | ARM | Malta | MLT^*^ |
| Austria | AUT | Monaco | MCO^*^ |
| Azerbaijan | AZE | Montenegro | MNE^*^ |
| Belarus | BLR | Netherlands | NLD |
| Belgium | BEL | Norway | NOR^*^ |
| Bosnia & Herzegovina | BIH | Poland | POL |
| Bulgaria | BGR | Portugal | PRT |
| Croatia | HRV | Moldova | MDA |
| Cyprus | CYP^*^ | Romania | ROU |
| Czechia | CZE | Russia | RUS |
| Denmark | DNK | San Marino | SMR^*^ |
| Estonia | EST^*^ | Serbia | SRB |
| Finland | FIN | Slovakia | SVK |
| France | FRA | Slovenia | SVN |
| Georgia | GEO | Spain | ESP |
| Germany | DEU | Sweden | SWE |
| Greece | GRC | Switzerland | CHE |
| Hungary | HUN | Tajikistan | TJK^*^ |
| Iceland | ISL^*^ | North Macedonia | MKD |
| Ireland | IRL | Turkey | TUR |
| Israel | ISR | Turkmenistan | TKM^‡^ |
| Italy | ITA | Ukraine | UKR |
| Kazakhstan | KAZ^†^ | United Kingdom | GBR |
| Kyrgyzstan | KGZ^†^ | Uzbekistan | UZB^*^ |
| Latvia | LVA |  |  |

### **Table S2. Country names and their corresponding World Bank country code.**

Captions: * - countries excluded while identifying optimal vaccine allocation strategies due to data sparsity; † - countries removed due to significant changes in mortality case definition;^14^ ‡ - country removed as it had no COVID-19 mortality data in *Our World in Data* at the time of this study.^13^

## **1.3 Linking stringency indices to community mobility**

| Symbol | Values | Notes |
| --- | --- | --- |
| day of week | Nominal categorical values, 1-7 | To capture within week variability. Work-related variability, for example, vary tremendously depending on the day of week |
| 1 | country | Country code | To capture country-specific random effects |
| setting | Type of mobility | Type of mobility, one of “retail”, “transit”, “grocery”, and “work”. |
| setting * day of week | The interaction term between the type of mobility and day of week | To capture the interaction between type of mobility and day of the week. |
| setting * month of year | The interaction term between the type of mobility and month of year | To capture variable specific mobility seasonality. This has similar problems with using day of year as a predictor - 2020 has not fully elapsed yet so we aren’t sure what happens in Jan or Dec. In this study, we assume Dec to be like Nov, and Jan to be like Feb. |
| stringency index^ | Government stringency index describing the intensity of COVID-19 related non-pharmaceutical interventions | To capture the large decrease in early 2020. Without adjusting for the stringency index, the early year mobility for 2021 and 2020 may be artificially pulled lower. |

### **Table S3. Covariates table for the general additive model (GAM) used in mobility projection (from March 2021 to December 2022)**

**Caption:**

Formula: Mobility ~ day of week + (1 | country) + setting * day of week + mobility setting * month of year + stringency index. Based on COVID-19 related non-pharmaceutical interventions recorded in Oxford COVID-19 Government Response Tracker, the stringency index represented the extent of containment and closure policies and public health information campaigns on the country level.^16^ The value of the stringency index ranges between 0 and 100, with 0 indicating the least stringent conditions, and 100 the most stringent conditions. We extracted stringency index data on 5 Mar 2021, on which complete records for our countries of interest are available before 22 Feb 2021. We assume over one year from 22 Feb 2021, as vaccines roll out, stringency indices gradually recover towards pre-pandemic levels. However, due to long-term behaviour and policy changes, we expect that stringency indices will never fully return to 0 in the time frame of this study. Thus, we assume stringency indices will return to 10 over 365 days, regardless of their positions on 22 Feb 2021, following a sigmoid function. After 22 Feb 2022, the stringency indices would stay at 10 to reflect any long-term changes COVID-19 policies have on human behaviours. We impute the mobility for countries without mobility data by taking an average of the geographic neighbours.

## **1.4 Current country-specific stringency indices**

| **Countries** | **Min** | **1st Quarter** | **Median** | **3rd Quarter** | **Max** |
| --- | --- | --- | --- | --- | --- |
| All available (n = 50) | 22 | 54 | 61 | 71 | 88 |
| Countries with fitted models  (n = 38) | 28 | 56 | 68 | 72 | 88 |

### **Table S4. Stringency indices on at the end of the observation window (i.e., 22 Feb 2021)**

## **1.5 Degree of missingness in mobility data and stringency index**

| **Countries** | **Number of Countries with Community Mobility Report** | **Number of Countries with Stringency Indices** |
| --- | --- | --- |
| All (n = 53) | 42 | 50 |
| Countries with fitted models  (n = 38) | 35 | 36 |

### **Table S5. Missingness in community mobility reports and stringency indices.**

Caption: Of all countries we fitted, Albania (ALB), Armenia (ARM), and Azerbaijan (AZE) do not appear in community mobility reports; Armenia (ARM) and North Macedonia (MKD) cannot be found in the stringency index database.

## **1.6 Proportions of countries by optimal vaccine prioritization strategies**

| Roll-out Scenario | Decision-making Metrics | Proportion of countries with fitted models with this optimal vaccine prioritisation strategy (n = 38) | | | |
| --- | --- | --- | --- | --- | --- |
|  |  | V+ | V20 | V60 | V75 |
| R1 | Deaths | 0.0263 | 0.1842 | 0.1316 | **0.6579** |
| R1 | Cases | 0.0000 | **1.0000** | 0.0000 | 0.0000 |
| R1 | Adj. Life Expectancy | 0.0263 | 0.3684 | **0.5526** | 0.0526 |
| R1 | Quality Adj. Life Years | 0.0526 | 0.3947 | **0.5000** | 0.0526 |
| R1 | Human Capital | 0.0263 | 0.3421 | **0.6053** | 0.0263 |
| R2 | Deaths | 0.2105 | **0.3158** | 0.2368 | 0.2368 |
| R2 | Cases | 0.1053 | **0.8947** | 0.0000 | 0.0000 |
| R2 | Quality Adj. Life Years | 0.2895 | **0.5263** | 0.1842 | 0.0000 |
| R2 | Adj. Life Expectancy | 0.2632 | **0.5789** | 0.1579 | 0.0000 |
| R2 | Human Capital | 0.2632 | **0.4737** | 0.2632 | 0.0000 |
| R3 | Deaths | 0.3421 | 0.1579 | **0.4474** | 0.0526 |
| R3 | Cases | **0.4474** | 0.0526 | 0.2895 | 0.2105 |
| R3 | Adj. Life Expectancy | **0.3684** | 0.1579 | 0.3421 | 0.1316 |
| R3 | Quality Adj. Life Years | **0.3947** | 0.0789 | 0.4211 | 0.1053 |
| R3 | Human Capital | **0.3684** | 0.1579 | **0.3684** | 0.1053 |
| R4 | Deaths | 0.4474 | **0.5000** | 0.0263 | 0.0263 |
| R4 | Cases | **0.4737** | 0.1316 | 0.1316 | 0.2632 |
| R4 | Adj. Life Expectancy | **0.4737** | 0.4474 | 0.0789 | 0.0000 |
| R4 | Quality Adj. Life Years | **0.5000** | 0.4211 | 0.0789 | 0.0000 |
| R4 | Human Capital | **0.4737** | 0.4737 | 0.0526 | 0.0000 |

### **Table S6. Proportions of countries with specific optimal vaccine prioritization strategies.**

**Caption:** The denominator for these proportions is 38, the number of countries within the WHO European Region without data availability or sparsity issues.

## **1.7 Proportions of population by optimal vaccine prioritization strategies**

| Roll-out Scenario | Decision-making Metrics | Proportion of population in countries with fitted models with this optimal vaccine prioritisation strategy (n = 848,407) | | | |
| --- | --- | --- | --- | --- | --- |
|  |  | V+ | V20 | V60 | V75 |
| R1 | Deaths | 0.0126 | 0.1521 | 0.0499 | **0.7854** |
| R1 | Cases | 0 | **1** | 0 | 0 |
| R1 | Adj. Life Expectancy | 0.0126 | 0.2454 | **0.7362** | 0.0057 |
| R1 | Quality Adj. Life Years | 0.132 | 0.2581 | **0.6042** | 0.0057 |
| R1 | Human Capital | 0.0126 | 0.2331 | **0.752** | 0.0022 |
| R2 | Deaths | 0.1721 | 0.1169 | 0.316 | **0.395** |
| R2 | Cases | 0.134 | **0.866** | 0 | 0 |
| R2 | Quality Adj. Life Years | 0.2419 | **0.4352** | 0.323 | 0 |
| R2 | Adj. Life Expectancy | 0.2467 | **0.5017** | 0.2517 | 0 |
| R2 | Human Capital | 0.2405 | **0.3988** | 0.3607 | 0 |
| R3 | Deaths | **0.474** | 0.1328 | 0.2911 | 0.1021 |
| R3 | Cases | **0.5175** | 0.0213 | 0.2189 | 0.2423 |
| R3 | Adj. Life Expectancy | **0.4788** | 0.1328 | 0.2404 | 0.148 |
| R3 | Quality Adj. Life Years | **0.4948** | 0.032 | 0.3455 | 0.1278 |
| R3 | Human Capital | **0.4788** | 0.1328 | 0.2606 | 0.1278 |
| R4 | Deaths | 0.2541 | **0.7401** | 0.0022 | 0.0035 |
| R4 | Cases | 0.2576 | **0.3194** | 0.2465 | 0.1764 |
| R4 | Adj. Life Expectancy | 0.2606 | **0.6568** | 0.0827 | 0 |
| R4 | Quality Adj. Life Years | 0.2641 | **0.6465** | 0.0895 | 0 |
| R4 | Human Capital | 0.2606 | **0.7337** | 0.0057 | 0 |

### **Table S7. Proportions of populations with specific optimal vaccine prioritization strategies.**

**Caption:** The denominator for these proportions is 848,407, the populations of countries within the WHO European Region without data availability or sparsity issues.

| Roll-out Strategy | Policy | Deaths | Cases | Adj. Life Expectancy | Quality Adj. Life Years | Human Capital |
| --- | --- | --- | --- | --- | --- | --- |
| R4 | V60 | 9 | 20 | 12 | 11 | 10 |
| **R4** | **V60a** | **17** | **5** | **10** | **16** | **18** |
| R4 | V60b | 12 | 13 | 10 | 11 | 10 |
| R4 | V75 | 11 | 13 | 10 | 11 | 11 |
| **R4** | **V75a** | **27** | **25** | **28** | **27** | **27** |
| R4 | V75b | 11 | 13 | 10 | 11 | 11 |

## **1.8 Vaccination strategies for adolescents**

### **Table S8. Counts of cases where a policy is ranked first in terms of optimising health and economic benefits (including ties)**

**Caption:** The denominator for these proportions is 38, the number of countries within the WHO European Region without data availability or sparsity issues. In the expansion (a), adolescents were vaccinated with the last group in the baseline strategy, i.e. younger adults; in the expansion (b), adolescents were vaccinated after the last group in the baseline strategy had reached their maximum uptake level. We only presented results under R4 because R1 and R2 do not involve enough vaccine doses for expansion (b) to reach adolescents; R3 reaches adolescents too late for expansion (b) for observation effects. We found that vaccinating adolescents would bring additional health and economic benefits and that vaccinating adolescents simultaneously with younger adults was more beneficial than vaccinating them after the maximum uptake level among younger adults have been reached.

## **1.9 TREND (Transparent Reporting of Evaluations with Nonrandomized Designs) statement checklist**

| **Paper Section/Topic** | **Item No.** | | **Descriptor** | Reported? | | Notes |
| --- | --- | --- | --- | --- | --- | --- |
|  |  |  |  |  | Pg # |  |
| **TITLE and ABSTRACT** | | | |  |  |  |
| Title and Abstract | 1 | | - Information on how units were allocated to interventions |  | Not Applicable | No intervention implemented, mentioned in title it’s a modelling study |
|  |  | | - Structured abstract recommended | ✓ | 1-2 |  |
|  |  | | - Information on target population or study sample | ✓ | 1-2 |  |
| **INTRODUCTION** | | | |  |  |  |
| Background | 2 | - Scientific background and explanation of rationale | | ✓ | 3 |  |
|  |  | - Theories used in designing behavioral interventions | |  | Not Applicable | No intervention involved |
| **METHODS** | | | |  |  |  |
| Participants | 3 | | - Eligibility criteria for participants, including criteria at different levels in recruitment/sampling plan (e.g., cities, clinics, subjects) | ✓ | 5 | We included criteria we used for including countries in analysis |
|  |  | | - Method of recruitment (e.g., referral, self-selection), including the sampling method if a systematic sampling plan was implemented | ✓ | 5 | While we have not done any explicit sampling in this study, we discussed the sampling methods/ biases used by our data sources |
|  |  | | - Recruitment setting |  | Not Applicable | No recruitment involved |
|  |  | | - Settings and locations where the data were collected | ✓ | 5, Supplemental Material |  |
| Interventions | 4 | | - Details of the interventions intended for each study condition and how and when they were actually administered, specifically including: |  |  | No intervention implemented. |
|  |  | | - - Content: what was given? | ✓ | 6-7 | In this mathematical modelling study, hypothetical vaccines of assumed characteristics are given to simulated population. |
|  |  | | - - Delivery method: how was the content given? |  | Not Applicable | No delivery occurred. |
|  |  | | - - Unit of delivery: how were subjects grouped during delivery? | ✓ | 6-7 | We used realistic vaccine roll-out scenarios and several different vaccine allocation strategies to allocate the hypothetical vaccines in the mathematical model. |
|  |  | | - - Deliverer: who delivered the intervention? |  | Not Applicable | No delivery occurred. |
|  |  | | - - Setting: where was the intervention delivered? |  | Not Applicable | No delivery occurred. |
|  |  | | - - Exposure quantity and duration: how many sessions or episodes or events were intended to be delivered? How long were they intended to last? | ✓ | 6-7 | One simulated vaccination event in the simulated population using the hypothetical vaccine. |
|  |  | | - - Time span: how long was it intended to take to deliver the intervention to each unit? | ✓ | 6-7 | We have four different country-level roll-out scenarios that ends on 2021-2022 respectively |
|  |  | | - - Activities to increase compliance or adherence (e.g., incentives) |  | Not Applicable |  |
| Objectives | 5 | | - Specific objectives and hypotheses | ✓ | 3-8 |  |
| Outcomes | 6 | | - Clearly defined primary and secondary outcome measures | ✓ | 7-8 | Five decision making metrics |
|  |  | | - Methods used to collect data and any methods used to enhance the quality of measurements | ✓ | 5, Supplemental Material |  |
|  |  | | - Information on validated instruments such as psychometric and biometric properties |  | Not Applicable | No existing instrument used. |
| Sample size | 7 | | - How sample size was determined and, when applicable, explanation of any interim analyses and stopping rules | ✓ | 3-5 | Sample size not explicitly calculated – it’s where data availability and quality allow for reasonable inference in the geographic region of interests |
| Assignment method | 8 | | - Unit of assignment (the unit being assigned to study condition, e.g., individual, group, community) | ✓ | 7-8 |  |
|  |  | | - Method used to assign units to study conditions, including details of any restriction (e.g., blocking, stratification, minimization) | ✓ | 3 | Policies are evaluated on the country level. |
|  |  | | - Inclusion of aspects employed to help minimize potential bias induced due to non-randomization (e.g., matching) |  | Not Applicable | No intervention implemented and no randomization involved. |
| Blinding (masking) | 9 | | - Whether or not participants, those administering the interventions, and those assessing the outcomes were blinded to study condition assignment; if so, statement regarding how the blinding was accomplished and how it was assessed | ✓ | Not Applicable | There are no participants. |
| Unit of Analysis | 10 | | - Description of the smallest unit that is being analysed to assess intervention effects (e.g., individual, group, or community) | ✓ | 7-8 | Country. |
|  |  | | - If the unit of analysis differs from the unit of assignment, the analytical method used to account for this (e.g., adjusting the standard error estimates by the design effect or using multilevel analysis) |  | Not Applicable | There is no difference between unit of analysis and unit of assignment – both country level. |
| Statistical methods | 11 | | - Statistical methods used to compare study groups for primary methods outcome(s), including complex methods for correlated data |  | 4-7 | The main analytical method in this study is compartmental transmission model (mathematical model). It doesn’t match the item exactly, but we attached the page number. |
|  |  |  | - Statistical methods used for additional analyses, such as subgroup analyses and adjusted analysis | ✓ | 7 | We have only used statistical model to analyse the secondary results. |
|  |  |  | - Methods for imputing missing data, if used | ✓ | Supplemental Material | We used linear interpolation for minor missingness in time series data (less than a week consecutively), spatial interpolation and linear regression models for major missingness (borrowing power from neighbours and other WHO/ Europe members) |
|  |  |  | - Statistical software or programs used | ✓ | 8 |  |
| **RESULTS** | | | |  |  |  |
| Participant flow | 12 | | - Flow of participants through each stage of the study: enrollment, assignment, allocation and intervention exposure, follow-up, analysis (a diagram is strongly recommended) |  | Not Applicable | No participants involved. |
|  |  | | - - Enrollment: the numbers of participants screened for eligibility, found to be eligible or not eligible, declined to be enrolled, and enrolled in the study |  | Not Applicable | No participants involved. |
|  |  | | - - Assignment: the numbers of participants assigned to a study condition | ✓ | 6-7 | Simulated populations are assigned to receive hypothetical vaccine products. |
|  |  | | - - Allocation and intervention exposure: the number of participants assigned to each study condition and the number of participants who received each intervention | ✓ | 5 | All countries eligible for analysis (i.e. not excluded in the fitting stage) have their population receive the hypothetical vaccines. |
|  |  | | - - Follow-up: the number of participants who completed the follow-up or did not complete the follow-up (i.e., lost to follow-up), by study condition |  | Not Applicable | No participants involved. |
|  |  | | - - Analysis: the number of participants included in or excluded from the main analysis, by study condition |  | Not Applicable | No participants involved. |
|  |  | | - Description of protocol deviations from study as planned, along with reasons |  | Not Applicable | No intervention implemented. |
| Recruitment | 13 | | - Dates defining the periods of recruitment and follow-up |  | Not Applicable | No participants involved and no intervention implemented. All presented as counterfactual using a mathematical model. |
| Baseline data | 14 | | - Baseline demographic and clinical characteristics of participants in each study condition | ✓ | 5 | Baseline and study population are the same. |
|  |  | | - Baseline characteristics for each study condition relevant to specific disease prevention research | ✓ | 5 | Baseline and study population are the same. |
|  |  | | - Baseline comparisons of those lost to follow-up and those retained, overall and by study condition |  | Not Applicable | No participants involved. |
|  |  | | - Comparison between study population at baseline and target population of interest |  | Not Applicable | Baseline and study population are the same. |
| Baseline equivalence | 15 | | - Data on study group equivalence at baseline and statistical methods used to control for baseline differences |  | Not applicable | Baseline and study population are the same. |
| Numbers analyzed | 16 | | - Number of participants (denominator) included in each analysis for each study condition, particularly when the denominators change for different outcomes; statement of the results in absolute numbers when feasible | ✓ | 9-13 | 38 countries are included in the full analysis. |
|  |  | | - Indication of whether the analysis strategy was “intention to treat” or, if not, description of how non-compliers were treated in the analyses |  | Not applicable | No intervention involved. |
| Outcomes and estimation | 17 | | - For each primary and secondary outcome, a summary of results for each estimation study condition, and the estimated effect size and a confidence interval to indicate the precision | ✓ | 9-13, supplemental material |  |
|  |  | | - Inclusion of null and negative findings | ✓ | Supplemental material | Relevant only for one of the secondary outcomes (country characteristics associated with the optimal vaccine allocation strategy) |
|  |  | | - Inclusion of results from testing pre-specified causal pathways through which the intervention was intended to operate, if any |  | Not Applicable | No intervention implemented. Statistical component in this study is not intended for any causal inference – but association seeking. |
| Ancillary analyses | 18 | | - Summary of other analyses performed, including subgroup or restricted analyses, indicating which are pre-specified or exploratory | ✓ | Supplemental material |  |
| Adverse events | 19 | | - Summary of all important adverse events or unintended effects in each study condition (including summary measures, effect size estimates, and confidence intervals) |  | Not applicable | No intervention implemented. |
| **DISCUSSION** | | | |  |  |  |
| Interpretation | 20 | | - Interpretation of the results, taking into account study hypotheses, sources of potential bias, imprecision of measures, multiplicative analyses, and other limitations or weaknesses of the study | ✓ | 13-15 |  |
|  |  | | - Discussion of results taking into account the mechanism by which the intervention was intended to work (causal pathways) or alternative mechanisms or explanations | ✓ | 13-15 | Presented where relevant. |
|  |  | | - Discussion of the success of and barriers to implementing the intervention, fidelity of implementation |  | Not applicable | No intervention implemented. |
|  |  | | - Discussion of research, programmatic, or policy implications | ✓ | 13-15 |  |
| Generalizability | 21 | | - Generalizability (external validity) of the trial findings, taking into account the study population, the characteristics of the intervention, length of follow-up, incentives, compliance rates, specific sites/settings involved in the study, and other contextual issues | ✓ | 13-15 | Presented where relevant, given no intervention implemented. |
| Overall evidence | 22 | | - General interpretation of the results in the context of current evidence and current theory | ✓ | 13-15 |  |

**Table S9. TREND statement checklist**

*From:*  Des Jarlais, D. C., Lyles, C., Crepaz, N., & the Trend Group (2004). Improving the reporting quality of nonrandomized evaluations of behavioral and public health interventions: The TREND statement. *American Journal of Public Health*, 94, 361-366. For more information, visit: <http://www.cdc.gov/trendstatement/>

# **Supplemental Figures**

## **2.1 Age pyramid in the WHO European Region**


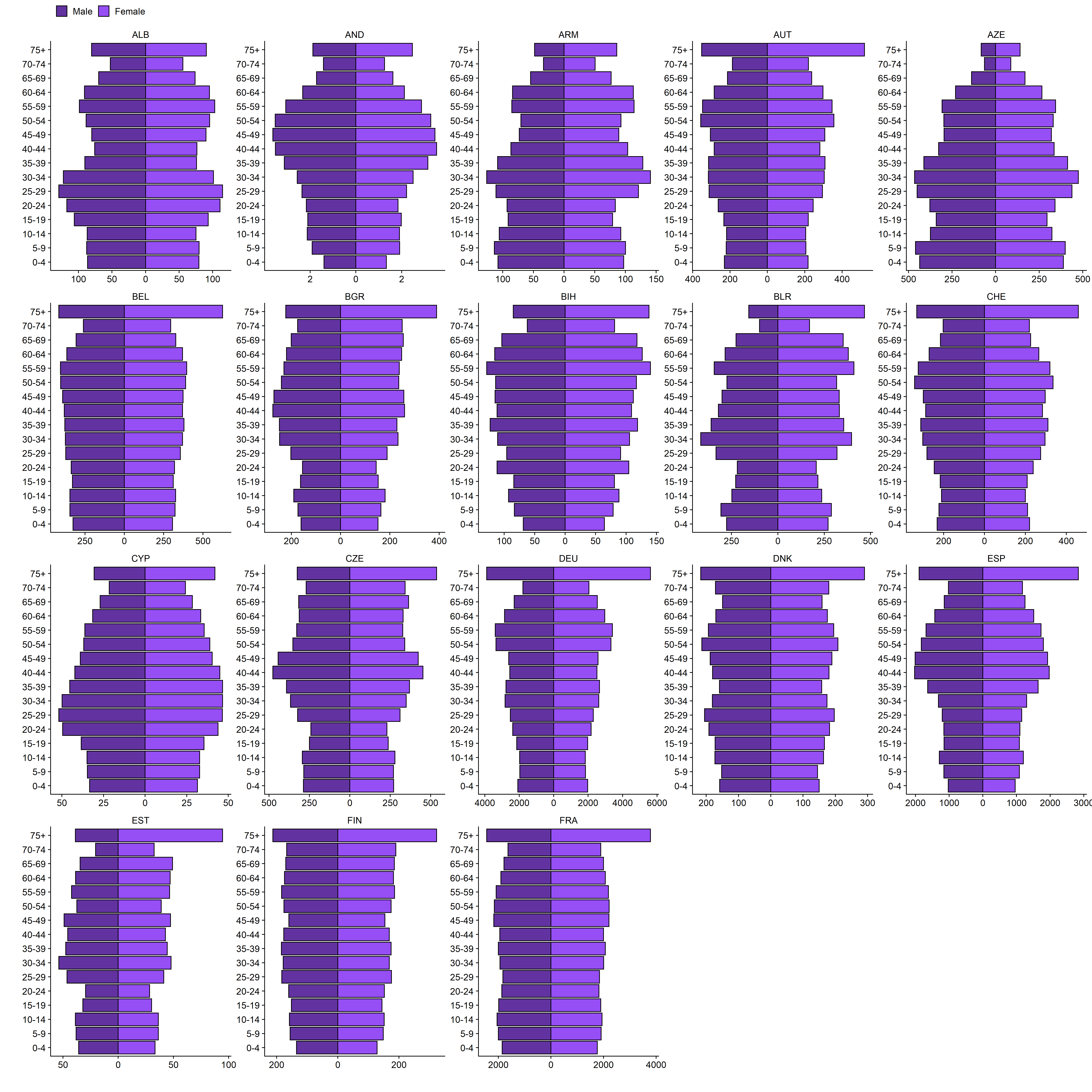


### **Figure S1. Population age pyramid by country.**

Data source: United Nations.^4^

Caption: Countries are labelled using their three-digit World Bank country codes.


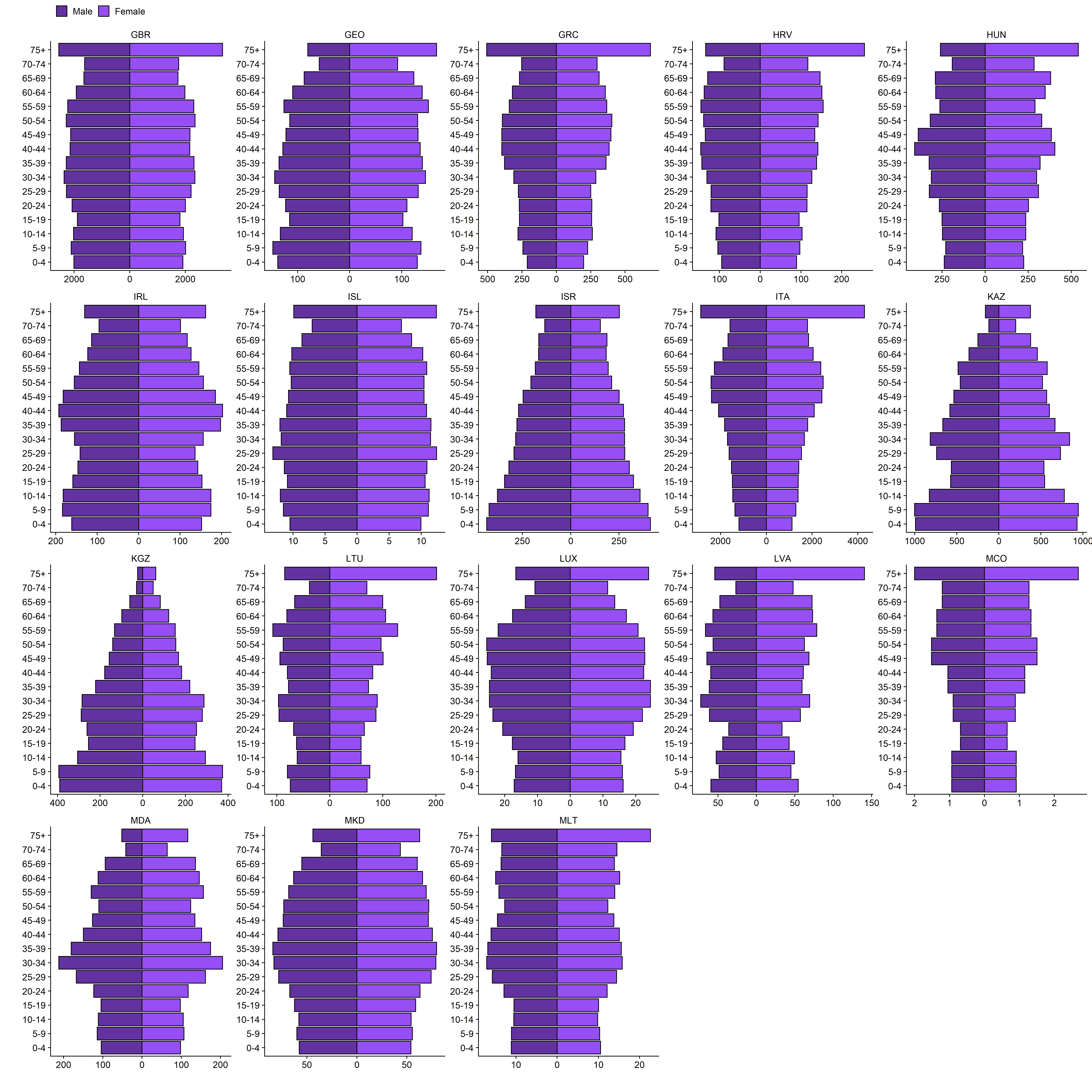
**Figure S1. Population age pyramid by country (continued).**


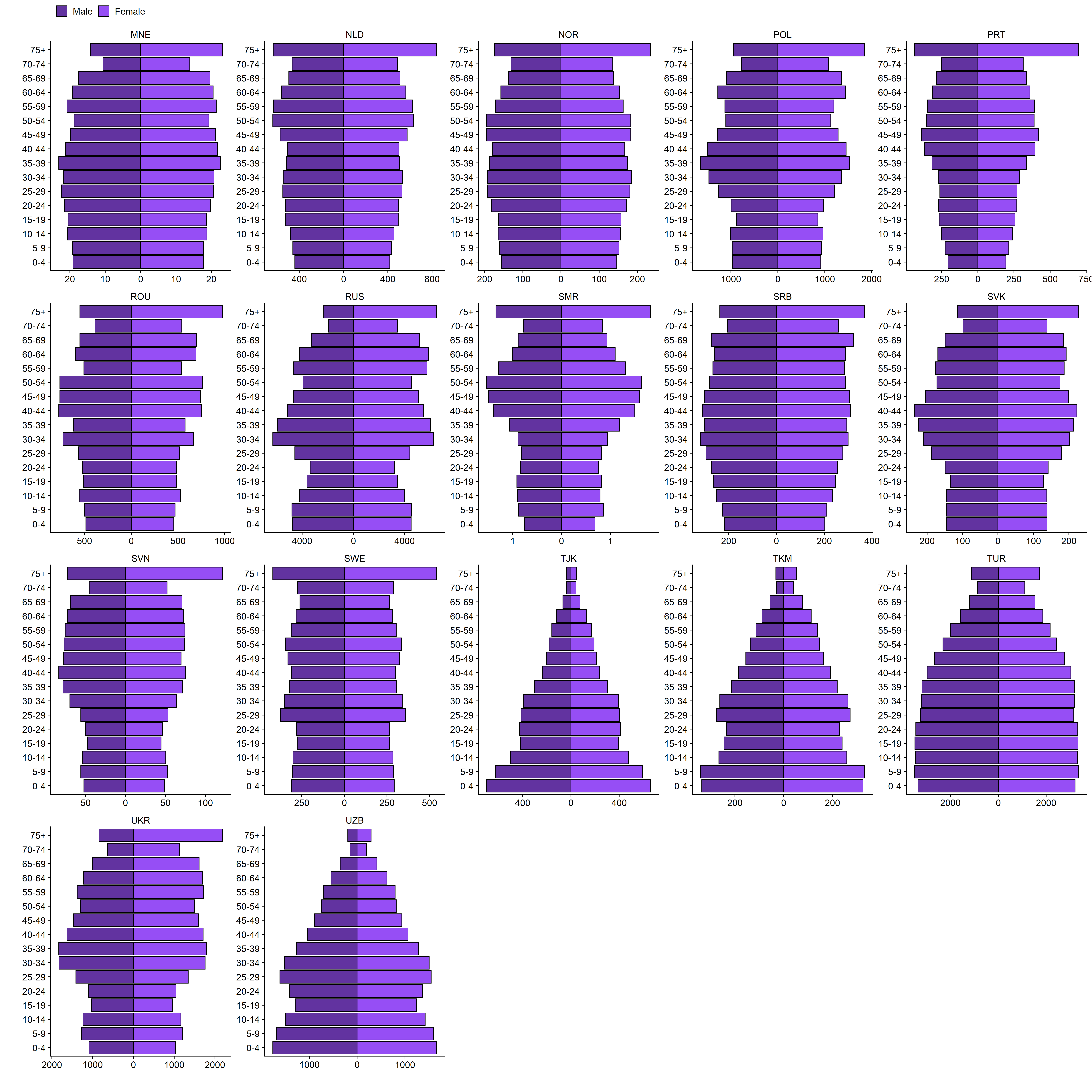


**Figure S1. Population age pyramid by country (continued).**

## **2.2 Results projecting stringency index into the future**


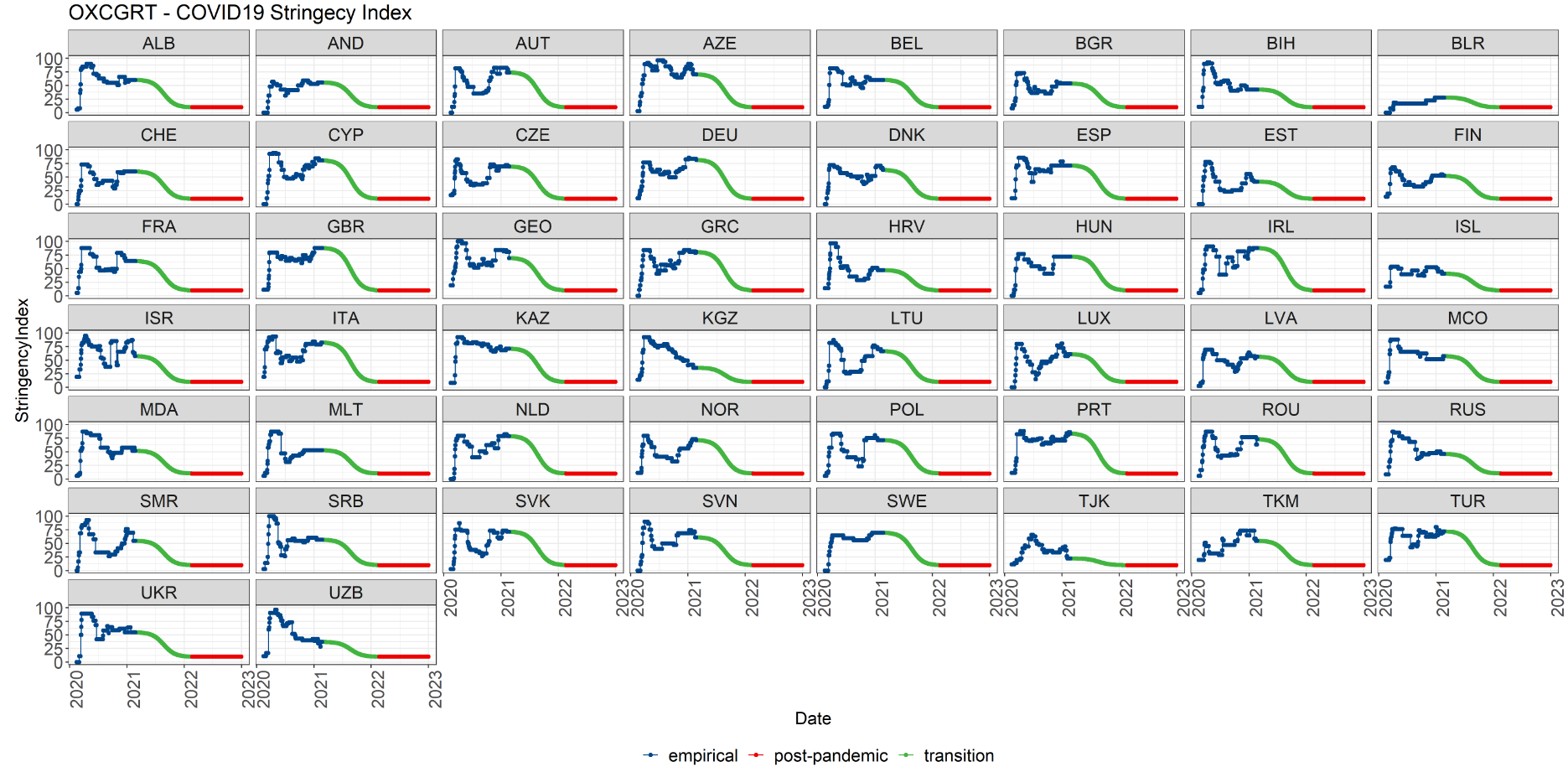


### **Figure S2. Stringency indices by country after incorporating the assumption on mobility recovery.**

**Caption:** Dark blue – empirical observations; green – transition phase; red – post-pandemic re-stabilised phase.

## **
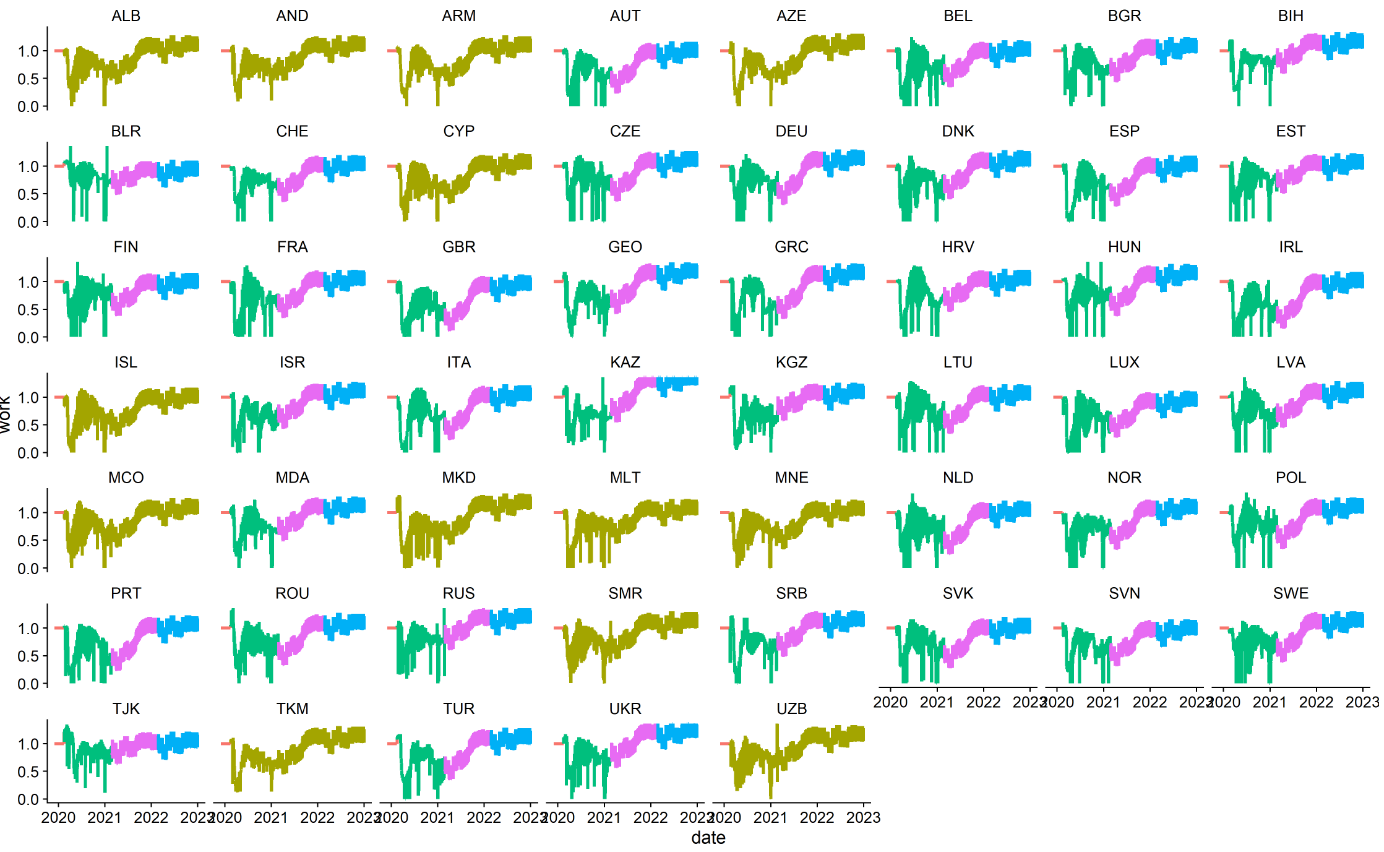
2.3. Projected population contacts – *work* setting**

### **Figure S3. Projected multipliers of daily contacts before December 2022 in the *work* setting**

**Caption:** Projected multipliers of baseline contact matrices capture the changes in population contact intensity. These multipliers were calculated using projected stringency indices, projected community mobility, and the relationship between daily contacts and community mobility defined by Davies et al.^5^ using UK data. Colours: coral = pre-pandemic baseline; dark yellow = imputed data for countries where community mobility data is not available; green = empirical observation; pink = transition phase between pandemic and post-pandemic phases; blue = post-pandemic phase.

## **2.4 Projected population contacts – *school* setting**
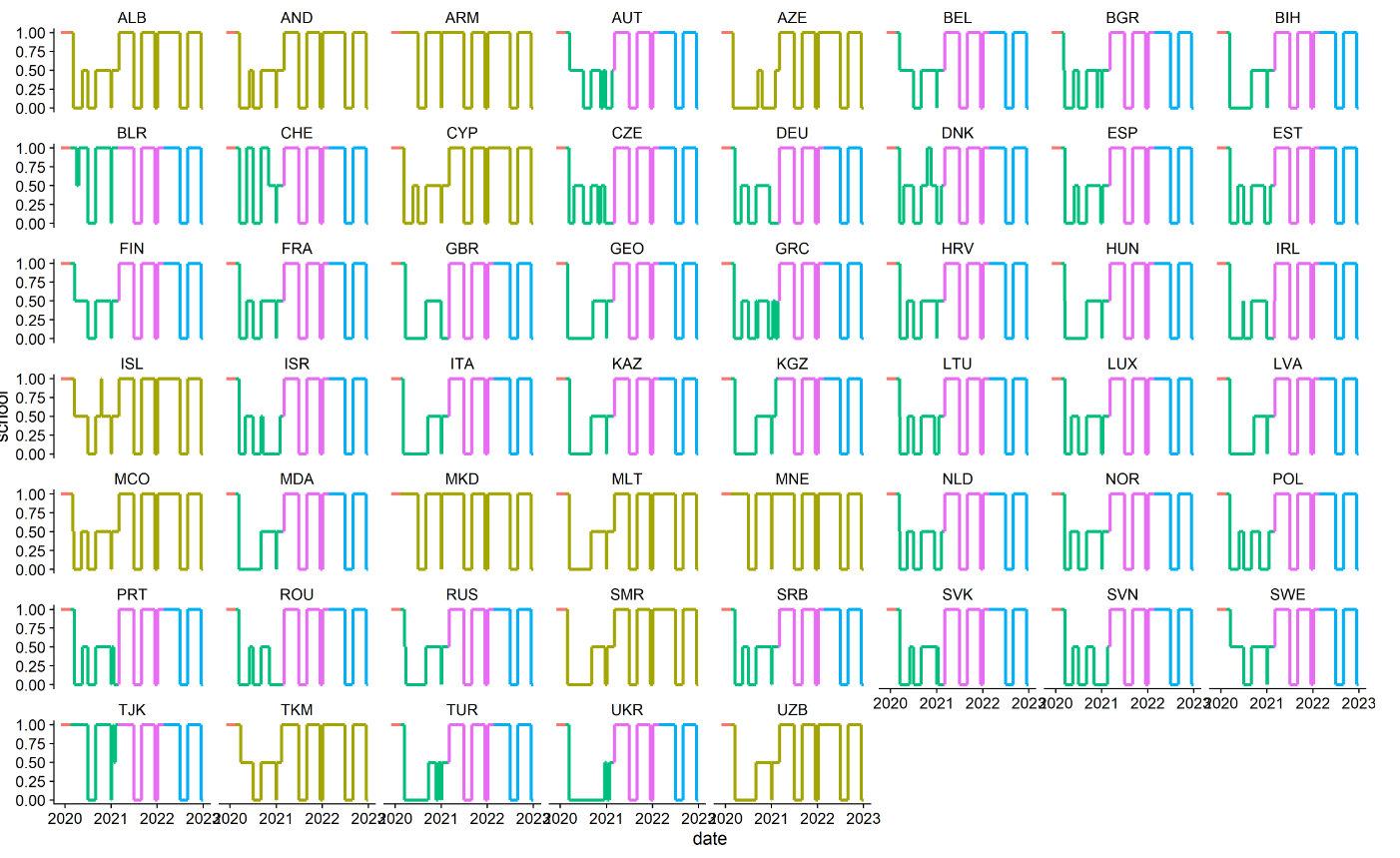


### **Figure S4. Projected multipliers of daily contacts before December 2022 in the *school* setting.**

**Caption:** Projected multipliers of baseline contact matrices captured the changes in population contact intensity. Colours: coral = pre-pandemic baseline; dark yellow = imputed data for countries where community mobility data is not available; green = empirical observation; pink = transition phase between pandemic and post-pandemic phases; blue = post-pandemic phase. During the period where empirical observation was available, the contacts in the *school* setting were modulated by the “school closure” variable (i.e. C1_school_closing) in the Oxford COVID-19 government response tracker. The variable C1_school_closing is ordinal: when C1_school_closing reached the highest value (i.e. 3, require closing all levels), the multiplier was set to 0 to indicate no school contacts; when C1_school_closing reached the lowest value (i.e. 0, require closing all levels), the multiplier was set to 1 to indicate *school*-based contacts were similar to pre-pandemic levels; when C1_school_closing was set to levels in between, the multiplier was assumed to be 0.5 to indicate an intermediate level of *school*-based contact intensity. During the transition between pandemic and post-pandemic phases and the post-pandemic phases, contacts in the *school* setting were only driven by the summer and winter holidays. The timing of summer and winter holidays varies in the European Union.^25^ Here, we broadly assume summer holidays to go for two months between July and August, and winter holidays to go for 3 weeks between mid-December to the first week of January. During school holidays, *school*-based contacts were set to 0.

## **
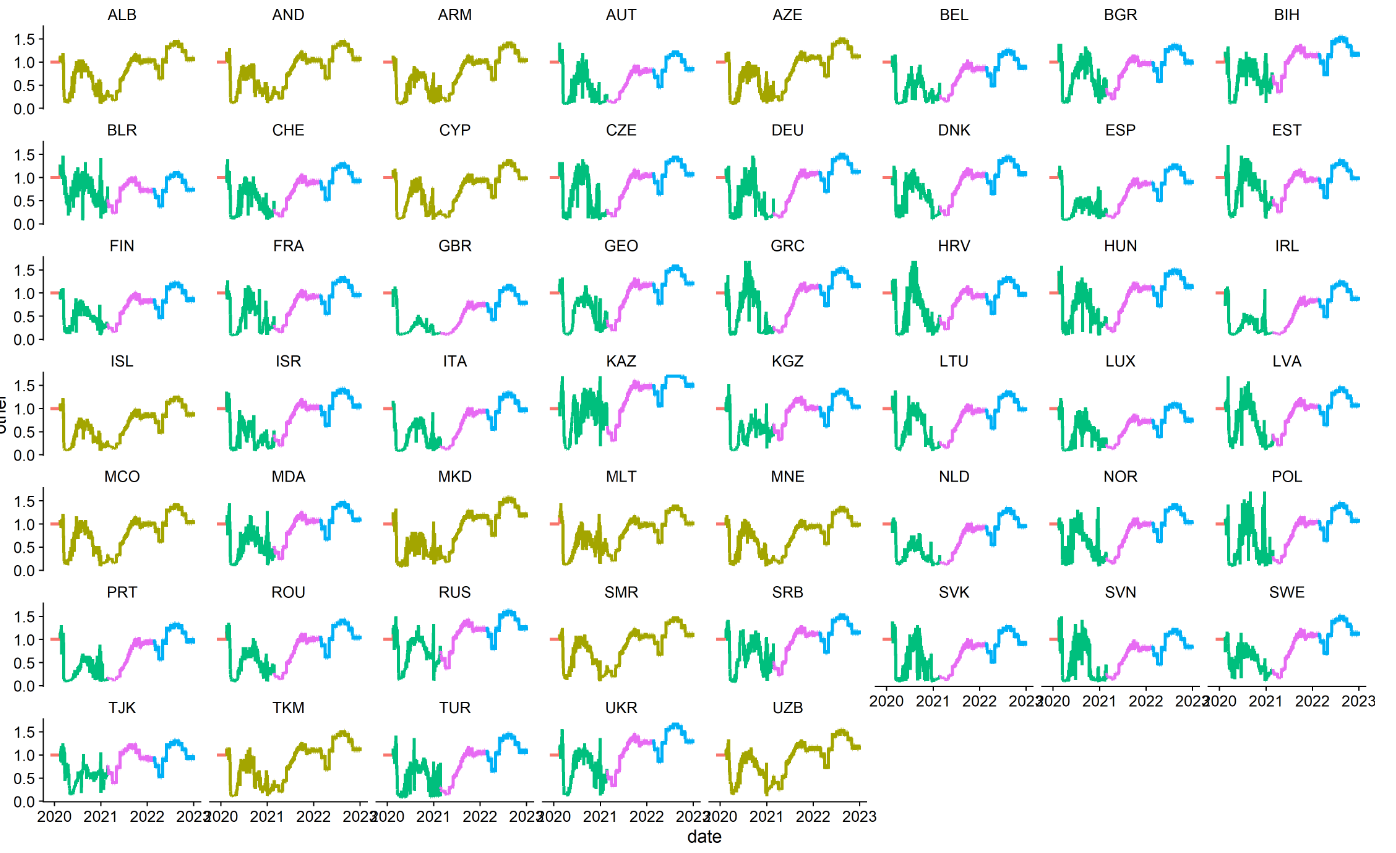
2.5 Projected population contacts – *others* setting**

### **Figure S5. Projected multipliers of daily contacts before December 2022 in the *others* setting.**

Caption: Projected multipliers of baseline contact matrices capture the changes in population contact intensity. These multipliers were calculated using projected stringency indices, projected community mobility, and the relationship between daily contacts and community mobility defined by Davies et al.^5^ using UK data. Colours: coral = pre-pandemic baseline; dark yellow = imputed data for countries where community mobility data is not available; green = empirical observation; pink = transition phase between pandemic and post-pandemic phases; blue = post-pandemic phase.

## **
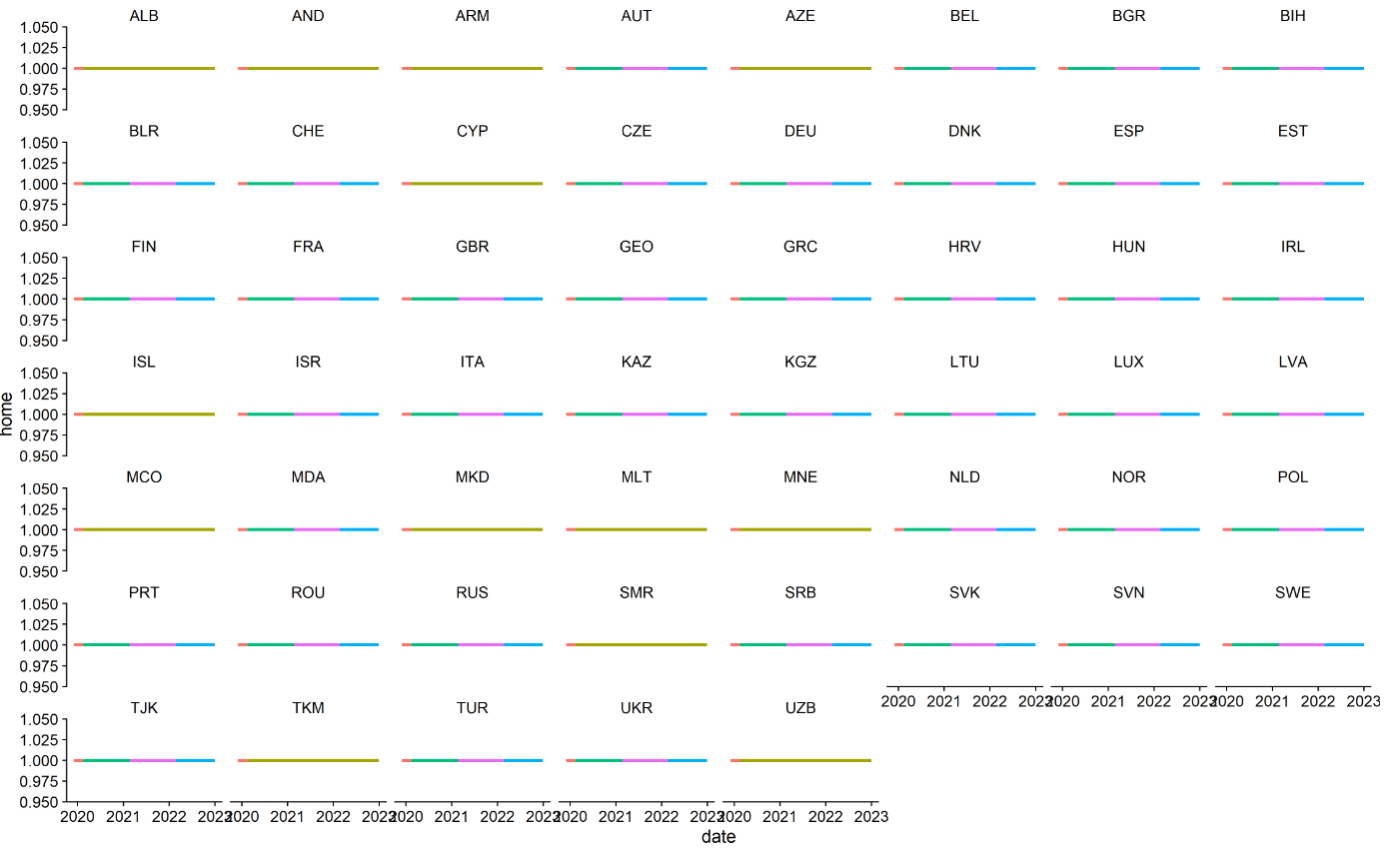
2.6 Projected population contacts – *home* setting**

### **Figure S6. Projected multipliers of daily contacts before December 2022 in the *home* setting.**

**Caption:** Projected multipliers of baseline contact matrices capture the changes in population contact intensity. Colours: coral = pre-pandemic baseline; dark yellow = imputed data for countries where community mobility data is not available; green = empirical observation; pink = transition phase between pandemic and post-pandemic phases; blue = post-pandemic phase. Home-based contacts are expected to stay constant in this study. The Google community mobility report showed increased time spent at home during the pandemic.^15^ We argue that this change does not affect transmission as both pandemic and pre-pandemic time and contacts at home are likely above the time and contacts required for transmission.^26^

## **
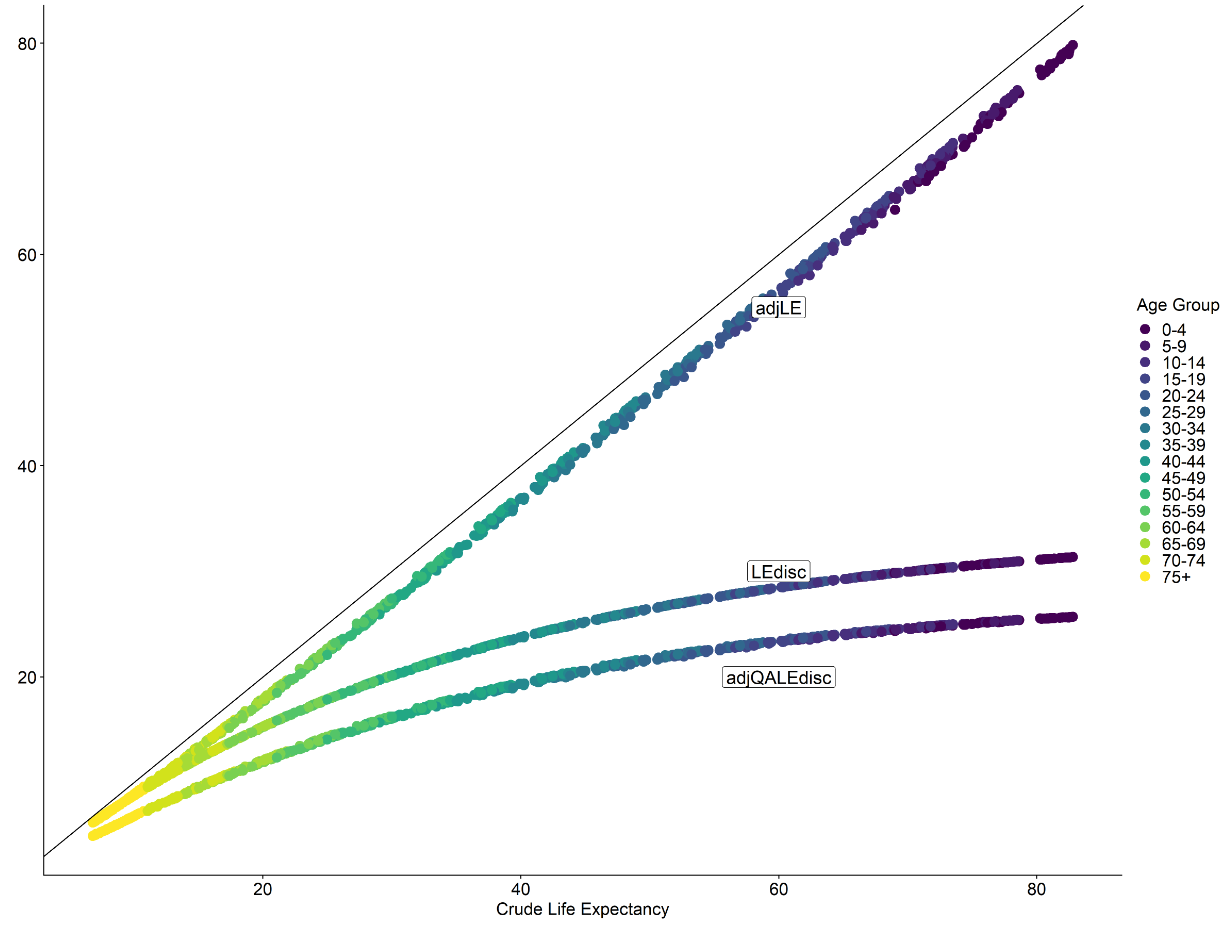
2.7 Numeric comparison between adjLE, LEdisc, and adjQALEdisc**

### **Figure S7. Numeric comparison between crude life expectancy and comorbidity adjusted life expectancy (adjLE), discounted life expectancy (LEdisc) and discounted comorbidity- and quality-adjusted life expectancy (adjQALEdisc).**

## **2.8 Values of Comorbidity adjusted life years**

###
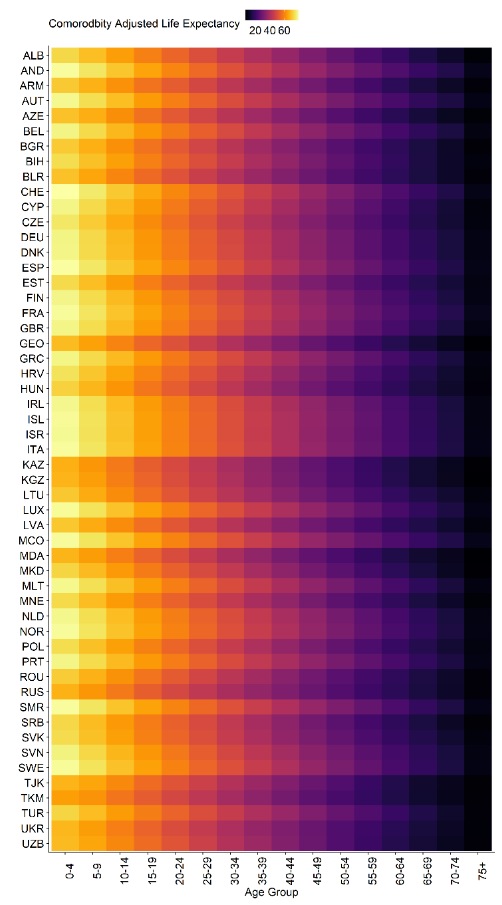
**Figure S8. Comorbidity adjusted life expectancy by age and by country**

## **
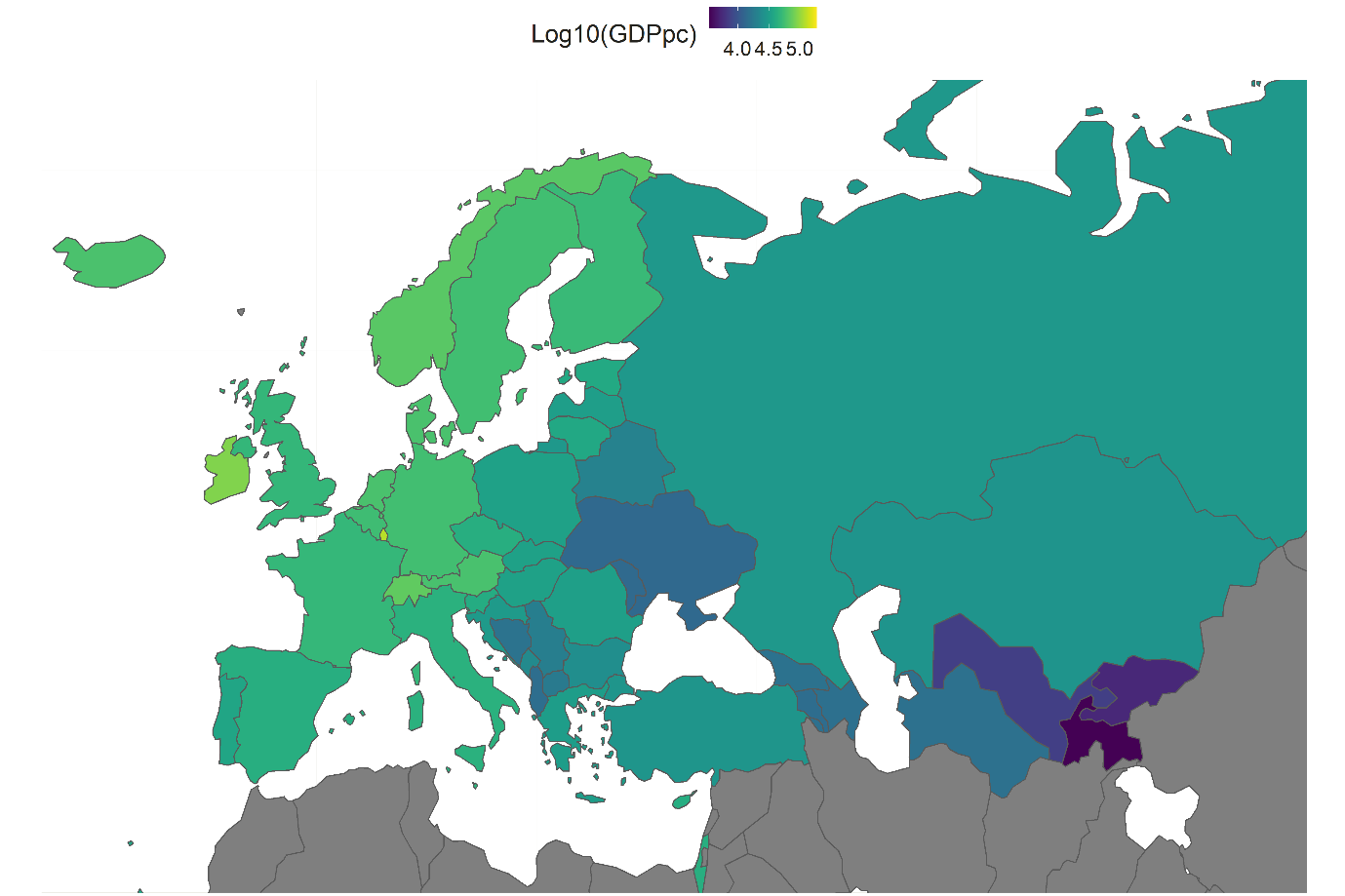
2.9 Values of GDP per capita**

### **Figure S9. Gross Domestic Production per capita (GDPpc, log-scaled) in the WHO European Region**

##
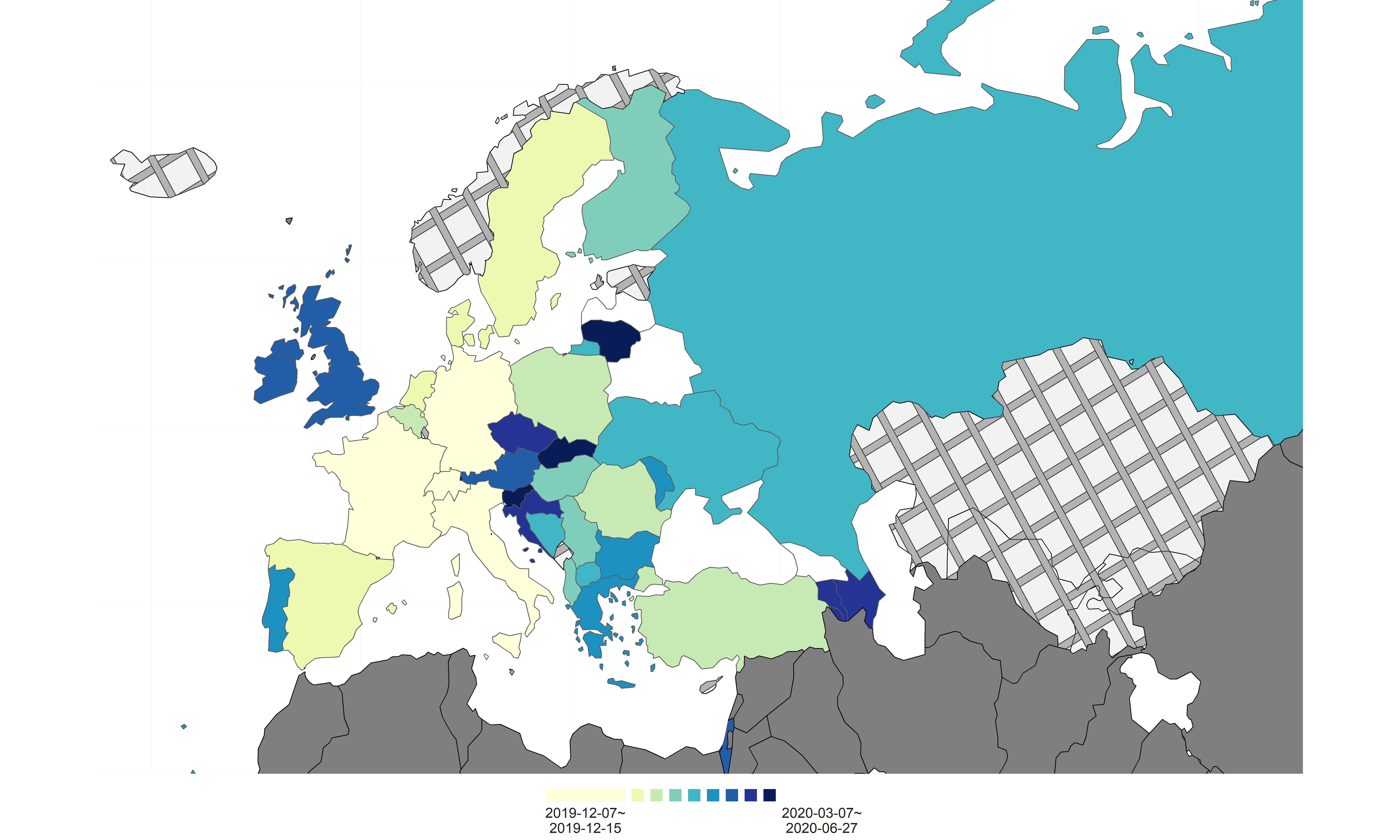
**[Two-variable fitting process] Estimated Infection Introduction Dates**

### **Figure S10. Fitted infection introduction dates in the WHO European Region.**

**Caption**: The underlying fitting structure involves two varying parameters - infection introduction date and the basic reproduction number. Country shapefiles are downloaded from Eurostat GISCO.^27^ Cross-hatched regions indicate countries where model fit was not achieved due to data availability and quality issues discussed in the main text.

##
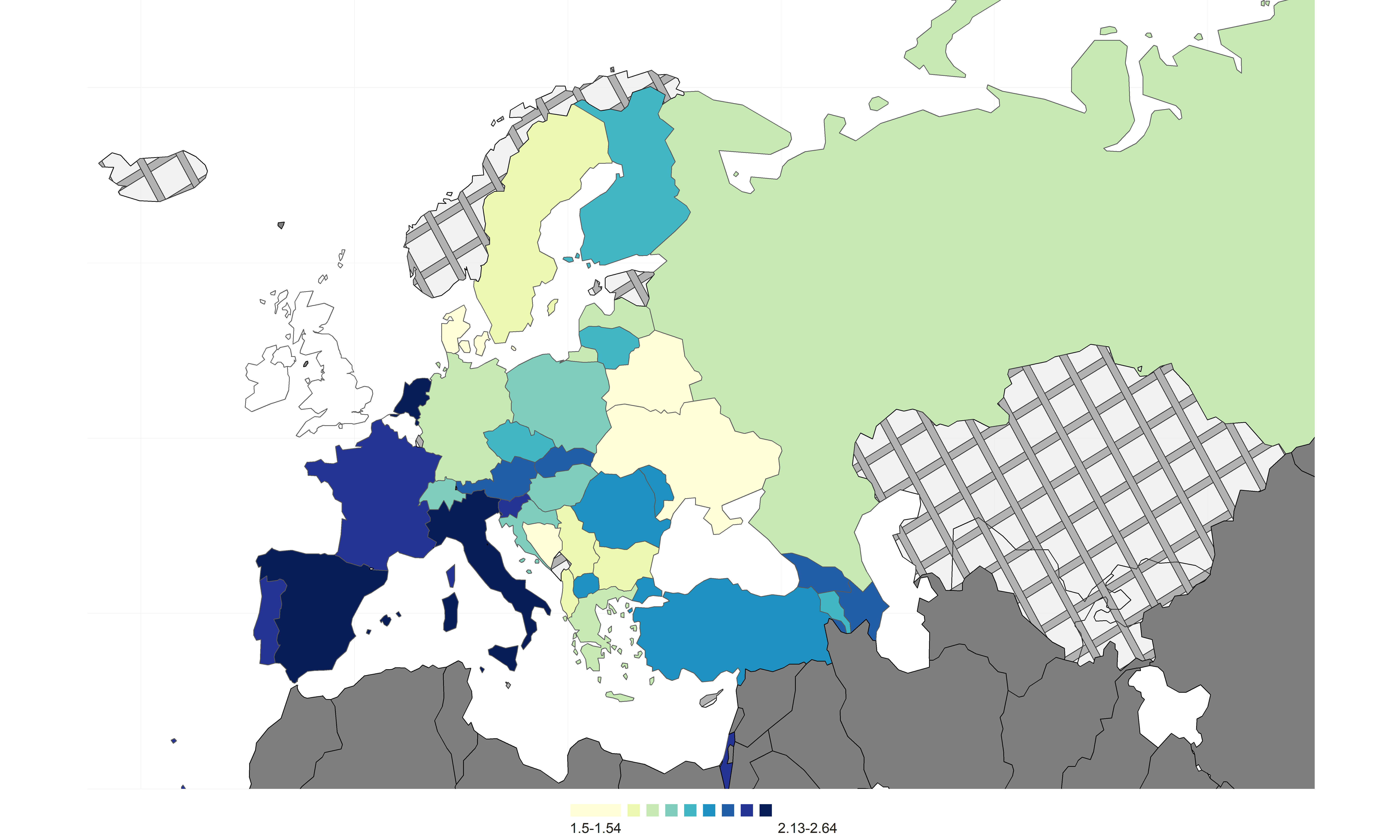
**[Two-variable fitting process] Estimated Infection Introduction Dates**

### **Figure S11. Fitted basic reproduction numbers in the WHO European Region.**

Caption: The underlying fitting structure involves two varying parameters - infection introduction date and the basic reproduction number. Country shapefiles are downloaded from Eurostat GISCO.^27^ Cross-hatched regions indicate countries where model fit was not achieved due to data availability and quality issues discussed in the main text.

## **2.12 Results of ordinal logistic regression exercise**

### **Figure S12. Coefficients and their corresponding 90% and 95% confidence interval in the ordinal logistic regression model.**
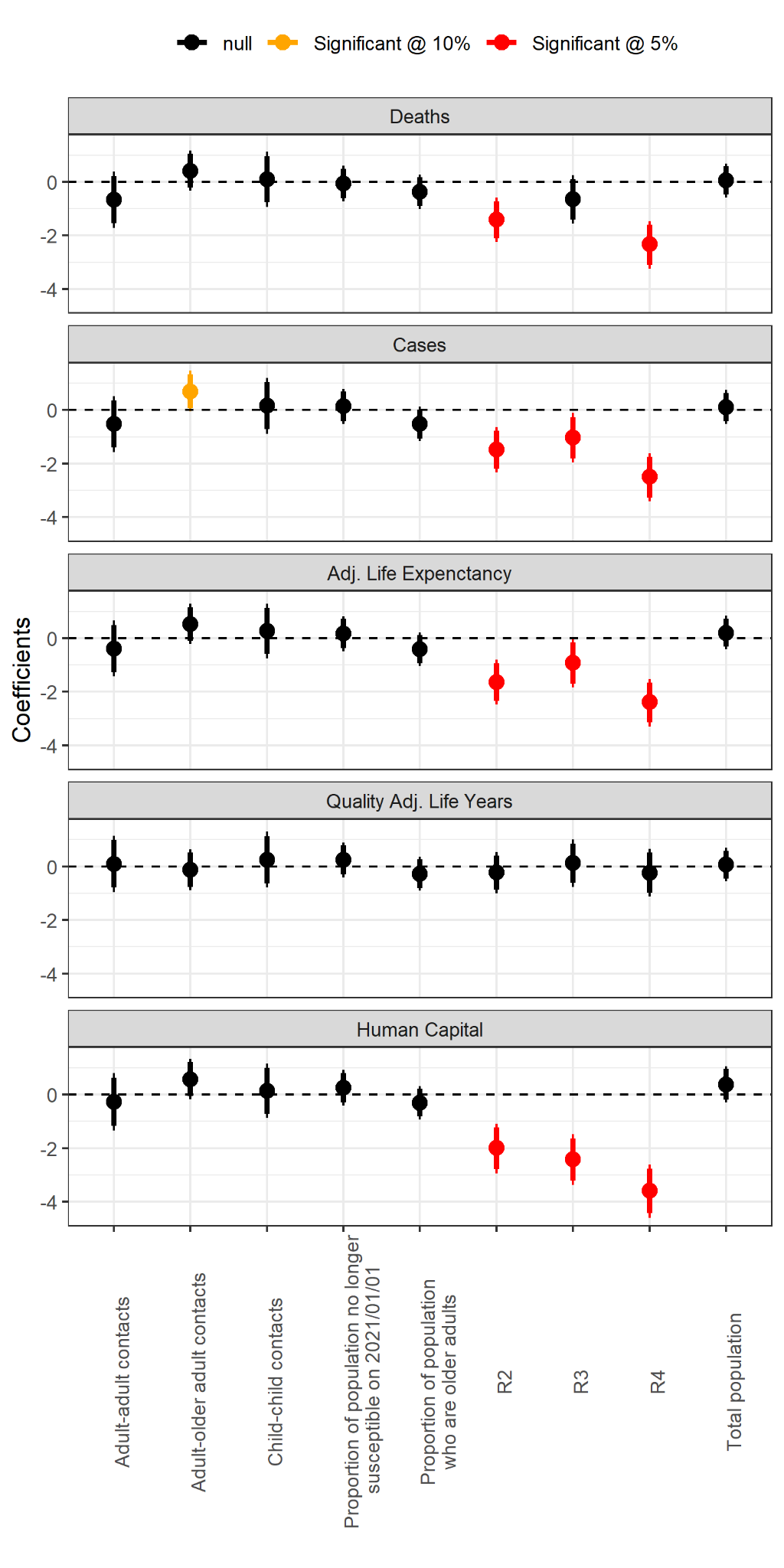


**Caption**: The sole dependent variable is the optimal vaccine prioritisation strategy identified. There are five groups of independent variables: (1) age-specific contact patterns, (2) vaccine rollout scenarios, (3) population size, (4) proportion of older adults, and (5) the proportion of individuals no longer susceptible to SARS-CoV-2 by 01 January 2021. Not all variables within each group were presented here - those with Pearson’s correlation larger than 0.4 were eliminated to avoid multicollinearity issues.

## **2.13 [Sensitivity analysis] Longer waning period for vaccine-induced immunity**


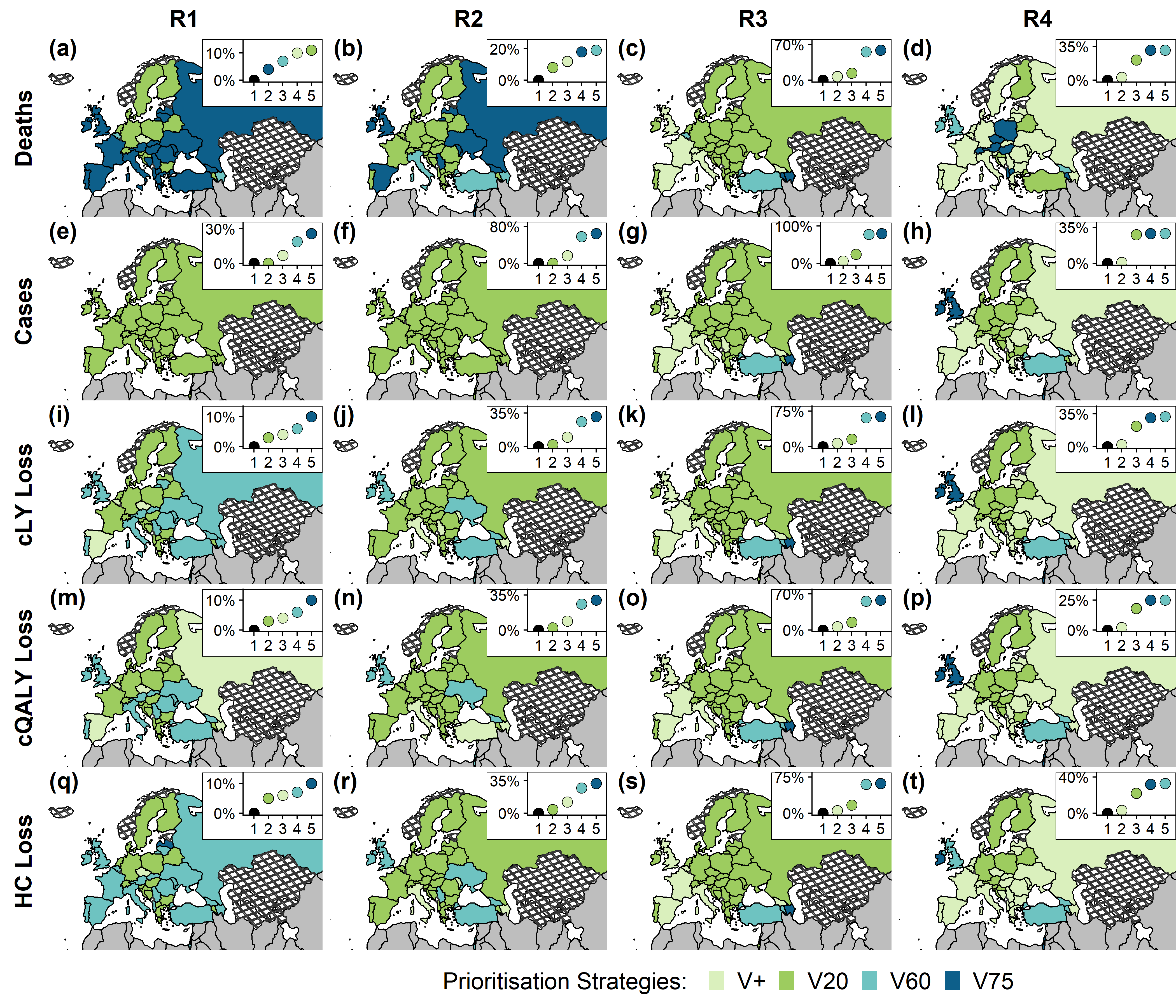
Figure S13. Optimal vaccine prioritisation strategies under different rollout scenarios and decision-making metrics using a longer vaccine waning period

**Caption:** The underlying fitting structure involves two varying parameters - infection introduction dates and the basic reproduction numbers. Vaccine-induced immunity is assumed to wane exponentially over 3 years (as opposed to 52 weeks, presented in the main text). Main panel - Optimal strategies across the WHO European Region identified using decision metrics of cumulative COVID-19 deaths, cases, and losses in comorbidity-adjusted life expectancy (cLE), comorbidity- and quality-adjusted life-years (cQALY), and human capital (HC). Inner panel - Comparing the use of a given prioritisation strategy across the WHO European Region against the use of country-specific optimal prioritisation strategies (indicated with black points). Country shapefiles are downloaded from Eurostat GISCO;^27^ countries marked by crosshatch patterns are those that were not included in the fitting stage.

## **2.14 [Sensitivity Analysis] VOC transmissibility adjustment**

### **Figure S14. Sensitivity analyses results showing changes in optimal vaccine prioritisation strategies using a pathogen that becomes 50% more transmissible on 15 April 2021.**

**Caption:** The denominator for these proportions is 38, the number of countries within the WHO European Region without data availability or sparsity issues. The 50% increase has been implemented on the pathogen transmissibility. The temporal cut off on 15 April 2021 was selected based on the observed Delta variant detection rates.^28^

## **2.15 [Sensitivity analysis] Underreporting**


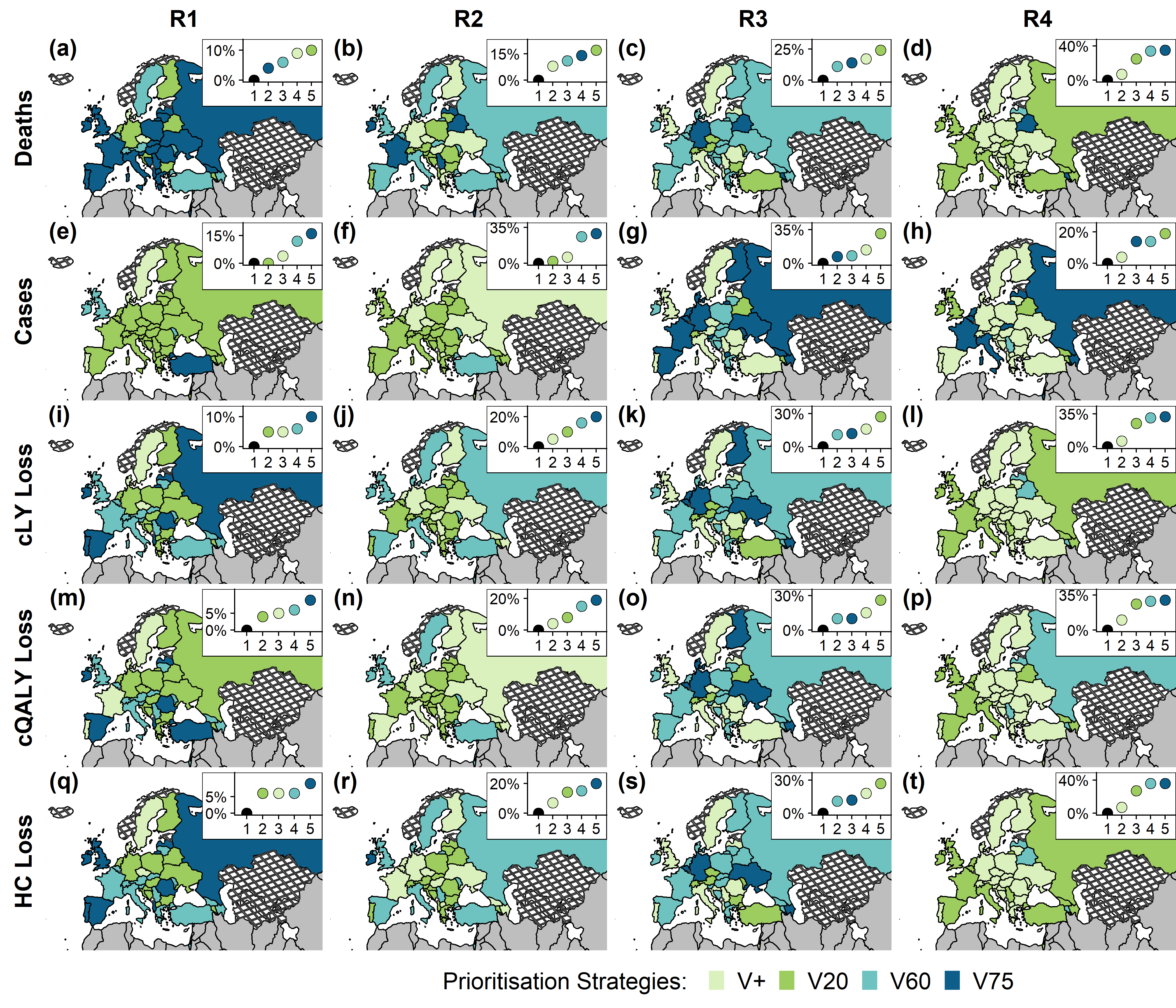
 Figure S15. Optimal vaccine prioritisation strategies under different roll-out scenarios and decision-making metrics considering underreporting

**Caption:** The underlying fitting structure involves three varying parameters - infection introduction dates, the basic reproduction numbers, and an underreporting probability. Main panel - Optimal strategies across the WHO European Region identified using decision metrics of cumulative COVID-19 deaths, cases, and losses in comorbidity-adjusted life expectancy (cLE), comorbidity- and quality-adjusted life-years (cQALY), and human capital (HC). Inner panel - Comparing the use of a given prioritisation strategy across the WHO European Region against the use of country-specific optimal prioritisation strategies (indicated with black points). Country shapefiles are downloaded from Eurostat GISCO;^27^ countries marked by crosshatch patterns are those that were not included in the fitting stage.

## **2.16 [Sensitivity analysis] Different decision time frames**

**
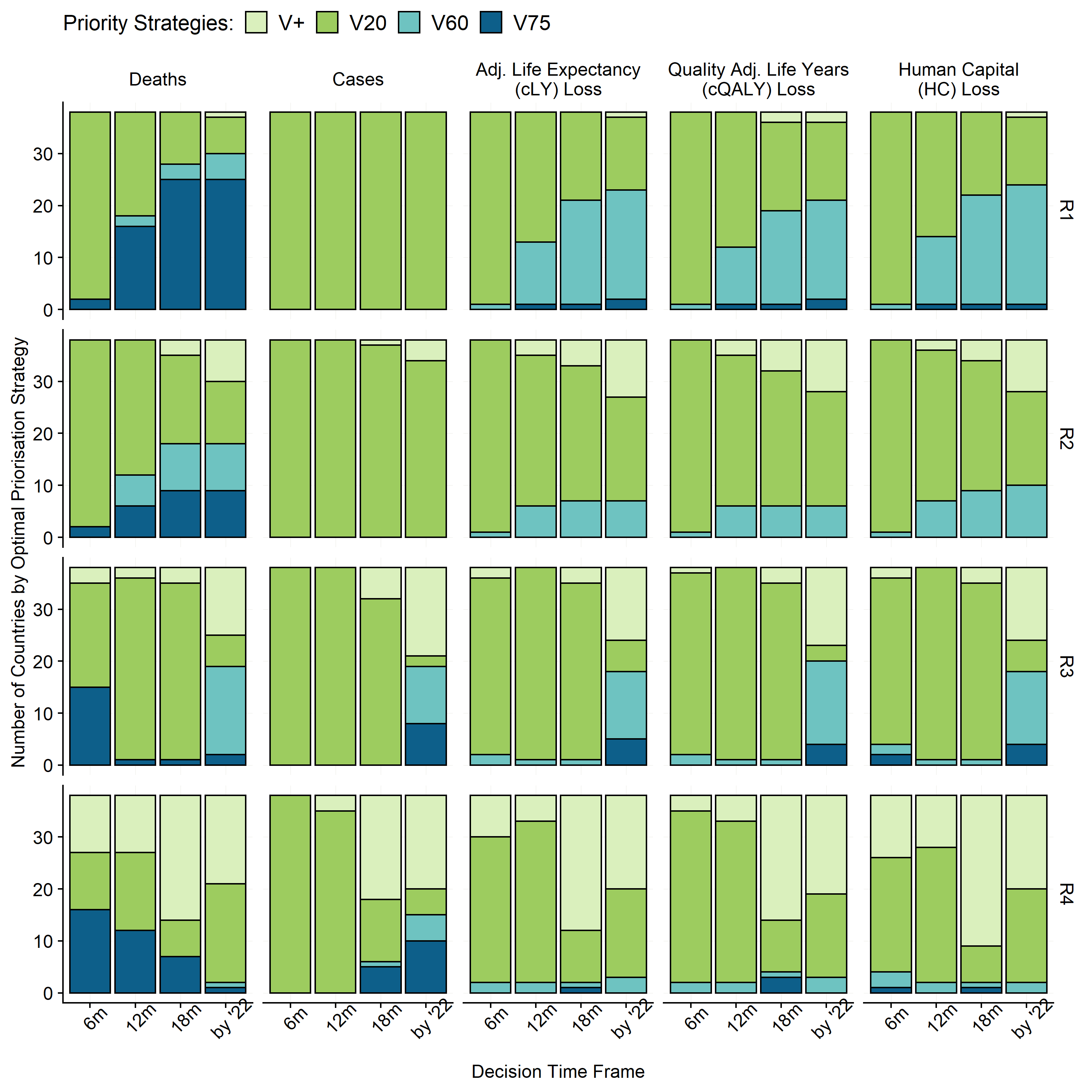
**

### **Figure S16. Optimal vaccine prioritisation strategies under different roll-out scenarios when decision-making metrics were summarised over different decision-making time frames.**

**Caption:** The results presented in these columns are each decision-making metric summarised between 01 January 2021 and 31 December 2022. Noticeably, the vaccination programs elapsed for different durations under different rollout scenarios. For R1 and R2, the vaccination campaigns were assumed to start on 01 March 2021; for R3 and R4, the vaccination campaigns were assumed to start on 01 January 2021. The rest represents results summarised over decision time frames. Among them, “6m”, “12m” and “18m” represents decision time frames ending on the 6th, 12th, and 18th months after the start of vaccination campaigns. In these columns, vaccine program lengths were the same across roll-out strategies.

## **2.17 [Sensitivity Analysis] “Lower uptake targets”**

### **Figure S17. Sensitivity analyses results showing changes in optimal vaccine prioritisation strategies using the “current condition” set of uptake parameters.**

**Caption:** The denominator for these proportions is 38, the number of countries within the WHO European Region without data availability or sparsity issues. Original optimal strategies are based on the baseline uptake parameters (older adults = 0.9, younger adults 0.7); “Outcome with slightly lower uptake” is based on “current condition” uptake parameters (older adults = 0.8, younger adults = 0.65). Number of countries on the diagonal axis are where optimal strategies has not changed because of slightly lower uptake thresholds

## **2.18 [Sensitivity Analysis] “Extremely low uptake targets”**

### **Table S18. Sensitivity analyses results showing changes in optimal vaccine prioritisation strategies using the “extremely low” set of uptake parameters.**

**Caption:** The denominator for these proportions is 38, the number of countries within the WHO European Region without data availability or sparsity issues. Original optimal strategies are based on the baseline uptake parameters (older adults = 0.9, younger adults 0.7); “Outcome with slightly lower uptake” is based on “current condition” uptake parameters (older adults = 0.6, younger adults = 0.45). Number of countries on the diagonal axis are where optimal strategies has not changed because of slightly lower uptake thresholds.

## **2.19 Country-specific vaccine-prioritisation strategies by different vaccine profiles under R2 and R3**





### **Figure S19. Optimal vaccine prioritisation strategies for different vaccine characteristics under R2 and R3.**

**Caption:** Optimal strategy for each country and vaccine profile while minimising mortality, morbidity, adjusted life expectancy (cLE), quality-adjusted life-years (cQALY), or human capital (HC) losses for 38 countries in the WHO European Region with fitted models. Countries are arranged in the order of the expected proportion of the population no longer susceptible to SARS-CoV-2 on 01 Jan 2021 (descending). Supplementary Table S2 is a reference table for country names and country codes (presented in this figure).

## **2.20 Country-specific vaccine-prioritisation strategies by different vaccine profiles under R1 and R4**


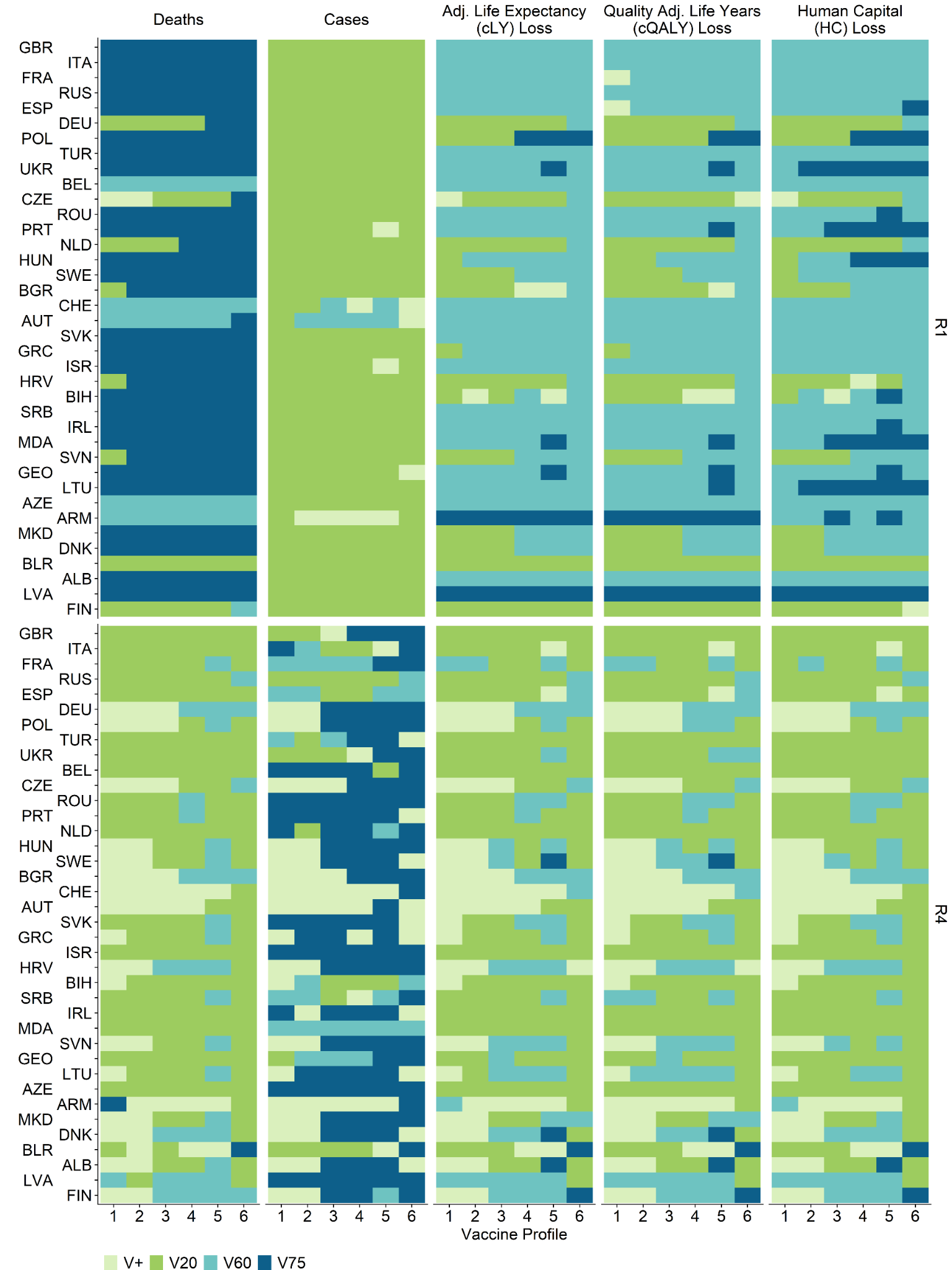


### **Figure S20. Optimal vaccine prioritisation strategies for different vaccine characteristics under R1 and R4.**

**Caption:** Optimal strategy for each country and vaccine profile while minimising mortality, morbidity, adjusted life expectancy (cLE), quality-adjusted life-years (cQALY), or human capital (HC) losses for 38 countries in the WHO European Region with fitted models. Countries are arranged in the order of the expected proportion of the population no longer susceptible to SARS-CoV-2 on 01 Jan 2021 (descending). Supplementary Table S2 is a reference table for country names and country codes (presented in this figure).

# **3. Supplemental Methods**

## **3.1 Algorithmic details of the transmission model**

### **3.1.1 Equations and Syntax**

The underlying mathematical transmission model used in this study has been previously described in detail elsewhere.^7^ We provide the mathematical equations used to capture the population dynamics:

| $S_{i}(t)$ | Susceptible individuals among age group *i* at time *t* |  |
| --- | --- | --- |
|  | $\frac{dS_{i}(t)}{dt}= -S_{i}\left( t \right)\cdot\lambda_{i}(t)-v_{i}\left( t \right)\cdot\frac{S_{i}\left( t \right)}{N_{i}}+R_{i}\left( t \right)\cdot\omega_{n}+V_{i}\left( t \right)\cdot\omega_{v}$ | (1) |
| $V_{i}(t)$ | Vaccinated individuals among age group *i* at time *t* |  |
|  | $\frac{dV_{i}(t)}{dt}\boldsymbol{=}{- V_{i}\left( t \right)\cdot\lambda_{i}(t)\cdot(1-e)+ v}_{i}\left( t \right)\cdot\frac{S_{i}\left( t \right)}{N_{i}}-V_{i}\left( t \right)\cdot\omega_{v}$ | (2) |
| $E_{i}(t)$ | Exposed individuals among age group *i* at time *t* |  |
|  | $\frac{dE_{i}(t)}{dt} = S_{i}\left( t \right)\cdot\lambda_{i}(t)+ V_{i}\left( t \right)\cdot\lambda_{i}(t)\cdot(1-e)(1-p_{a})-E_{i}\left( t \right)\cdot\sigma$ | (3) |
| $E_{vi}(t)$ | Vaccinated and Exposed individuals among age group *i* at time *t* who will only progress as subclinical infections |  |
|  | $\frac{dE_{vi}(t)}{dt}=V_{i}\left( t \right)\cdot\lambda_{i}(t)\cdot(1-e){\cdot p}_{a}-E_{vi}\left( t \right)\cdot\sigma$ | (4) |
| $I_{pi}(t)$ | Pre-clinical and infectious individuals among age group *i* at time *t* |  |
|  | $\frac{dI_{pi}(t)}{dt}= E_{i}\left( t \right)\cdot\sigma\cdot y_{i}- I_{pi}\left( t \right)\cdot\gamma_{p}$ | (5) |
| $I_{si}(t)$ | Subclinical and infectious individuals among age group *i* at time *t* |  |
|  | $\frac{dI_{si}(t)}{dt}= E_{i}\left( t \right)\cdot\sigma\cdot{(1-y}_{i})+E_{vi}\left( t \right)\cdot\sigma- I_{si}\left( t \right)\cdot\gamma_{s}$ | (6) |
| $I_{ci}\left( t \right)$ | Clinical and infectious individuals among age group *i* at time *t* |  |
|  | $\frac{dI_{ci}\left( t \right)}{dt}= I_{pi}\left( t \right)\cdot\gamma_{p}- I_{ci}\left( t \right)\cdot\gamma_{c}$ | (7) |
| $R_{i}(t)$ | Recovered individuals among age group *i* at time *t* |  |
|  | $\frac{dR_{i}(t)}{dt}= I_{ci}\left( t \right)\cdot\gamma_{c}+ I_{si}\left( t \right)\cdot\gamma_{s}- R_{i}\left( t \right)\cdot\omega_{n}$ | (8) |

Where:

| Variable Name | Definition and notes |
| --- | --- |
| $\lambda_{i}(t)$ | Force of infection $\lambda_{i}\left( t \right)=u_{i}\cdot\sum_{j=1}^{J} C_{i,j,t}\cdot\frac{(I_{pj}(t)+I_{cj}(t)+{f\cdot I}_{sj}(t))}{N_{j}}$ Where *j* depicts age group, *J* is 16, *f* is the relative infectiousness of subclinical individuals compared to pre-clinical and clinical individuals (i.e. 50%), and $u_{i}$ is susceptibility. |
| $v_{i}\left( t \right)$ | Number of vaccines allocated |
| $\omega_{n}$ | Natural immunity waning rate; natural immunity duration is thus 1/$\omega_{n}.$ |
| $\omega_{v}$ | Vaccine-induced immunity waning rate; vaccine induced immunity duration is thus 1/$\omega_{v}$ |
| $e$ | Vaccine efficacy against infection |
| $y_{i}$ | Clinical fraction |
| $1-p_{a}$ | Proportion of breakthrough infections among vaccinated individuals that progress similarly to infections among unvaccinated individuals |
| $1/\sigma$ | Latent period |
| $1/\gamma_{p}$ | Duration of preclinical infectiousness |
| ${1/\gamma}_{s}$ | Duration of subclinical infectiousness |
| ${1/\gamma}_{c}$ | Duration of clinical infectiousness |
| $u_{i}$ | Susceptibility  The next generation matrix (NGM) is defined as (*i* and *j* are age groups): ${NGM}_{ij}=u_{i}\cdot c_{i,j,t}\cdot(y_{j}\cdot\left( \frac{1}{\gamma_{p}}+ {\frac{1}{\gamma}}_{c} \right)+(1-y_{j})\cdot{\frac{1}{\gamma}}_{s}\cdot f)$ The absolute dominant eigenvalue of the NGM is scaled to match the estimated basic reproduction number (R_0_). |

### **3.2 Calculating COVID-19 mortality from the mathematical model**

1. Infection to mortality delay function is assumed to be a gamma distribution with mean of 26 (days) and shape of 5. This probability density function is capped at 60 (days).^6^

$$P= Gamma(mean=26;shape=5)$$

2. The deaths occurred on day t due to infection on day t-d can be expressed as (where ${IFR}_{j}$ depicts infection fatality ratio among age group *j*)

$${D(t)}_{dj}= {E(t-d)}_{j}\cdot P(d)\cdot{IFR}_{j}$$

3. The total deaths occurred on day t can thus be expressed as

$$D(t)= \sum_{j=1}^{J} \sum_{d=1}^{60} {E(t-d)}_{i}\cdot P(d)\cdot{IFR}_{j}$$

### **3.3 Incorporating vaccine efficacy against disease observed into the transmission model**

Without vaccines, $n_{j}$ infections are associated with $m_{j}$ symptomatic cases (i.e., “diseases”:

$$m_{j}=n_{j}\cdot j$$

where $y_{j}$ is the clinical fraction and *j* specifies the age group. With vaccines:

$$m_{vj}=n_{j}\cdot(1-e)\cdot(1-p_{a}){\cdot y}_{j}$$

where in $e$ is the vaccine efficacy against infections and $1-p_{a}$ is the proportion of breakthrough infections that progress similarly to those who have never been vaccinated. The overall vaccine efficacy observed ($e_{d}$) is a combination of infections reduced and clinical cases averted (i.e., cases that would have progressed clinically but ends up progressing subclinically due to vaccine protection).

$$M= \sum_{j=1}^{J} m_{j};M_{v}=\sum_{j=1}^{J} m_{vj}$$

$$1-e_{d}= \frac{M_{v}}{M}=(1-e)\cdot(1-p_{a})$$

Thus,

$$p_{a}=\frac{1-e_{d}}{(1-e)}$$

$$1-p_{a}=\frac{e_{d}-e}{(1-e)}$$

which explains why $e_{d}$ is equal to or greater than $e$.

## **More details on health and economic impact metrics**

### **3.2.1 Comorbidity-adjusted life expectancy, comorbidity- and quality-adjusted life expectancy, and discounted life expectancy**

Data on life expectancies for each country in the WHO European Region were taken from the website of WHO.^29^ We adjusted the life expectancies for higher risks of death due to comorbidities in those who die from COVID-19 using a recently proposed method and assuming an increased risk of 50%.^30^ We also adjusted for health-related quality of life (HRQoL) by age using EQ-5D-3L population norms from the seven countries in Europe with a time-trade off value set available (i.e., Denmark, France, Germany, Italy, Netherlands, Spain, UK),^31^ which indicates the potential loss of health-related quality of life (HRQoL) due to death per country (as deaths are based on country-specific estimates). Lastly, we also accounted for time preferences using a discount rate of 3.0% for future years.^32^

### **3.2.2 QALY associated with COVID-19 morbidity**

For morbidity, we assumed for each non-hospitalised case a QALY loss equivalent to symptomatic episodes of pandemic influenza-like illness.^33^ Furthermore, we assumed that 10% of cases are hospitalised (based on raw data of hospitalisations to cases), losing 0.0201 QALYs for more than 2 months post-discharge.^34^ Of the hospitalised cases, 50% were assumed to survive treatment in intensive-care units,^35^ with an estimated longer-term impact of ICU survivors of 0.15 QALYs.^36,37^ Another 10% of non-hospitalised cases were assumed to suffer from post-acute symptoms (long COVID),^38^ for whom we assumed a similar impact comparable to ICU survivors of 0.15 QALYs lost. In total, each symptomatic case is thus assigned a health loss equivalent to 0.0307 QALYs.

### **3.2.3 GDP per capita used in the human capital approach**

For the human capital approach, we used the annual GDP per capita in international dollars (intl$) in 2019 (or 2018 if unavailable) from the World Bank, converted by purchasing power parity (PPP).^39^ In the absence of these data for Andorra and Monaco, we used their GDP per capita in current US$ (without PPP conversion) from 2019 and 2018, respectively. With the GDP being a country-level productivity measure, combined with the country-specific life expectancies we derive an estimate of the economic losses per country (Figure S4) to add dimension to the health impact.

## **3.3 Impact and Health Economic Metrics**

The three health-economic metrics can then be calculated using the following equations:

1. Comorbidity-adjusted life expectancy (cLE) loss =

age-specific COVID-19 mortality *

age-specific comorbidity-adjusted life expectancy (adjLE)

1. Comorbidity- and quality-adjusted life year (cQALY) loss =

age-specific discounted comorbidity- and quality- adjusted life

expectancy (adjQALEdisc) *

age-specific COVID-19 mortality +

mean QALY loss associated with COVID-19 morbidity *

COVID-19 symptomatic cases +

mean QALY loss associated with AEFI * Number of vaccines deployed *

AEFI occurrence probability

1. Human Capital (HC) loss =

age-specific discounted life expectancy (LEdisc) *

age-specific COVID-19 mortality * GDP per capita**3.4 [Sensitivity analysis] Vaccine Uptake**

Vaccine availability (supply conditions) and uptake (the public willingness to be vaccinated) will both affect COVID-19 vaccine roll-out efforts. In the main analysis of this study, we assumed that 90% of those above 60 years of age and 70% among those between 20 and 59 were willing to be vaccinated. These values are in line with optimistic roll-out objects and intended uptake observed in the WHO European Region.^21,40^ We additionally referenced the WHO/ Europe COVID-19 Vaccine Programme Monitor, which showed that these uptake values are achievable in the WHO European Region based on data from countries with R3-R4 roll-out conditions.^41^ We tested two pairs of vaccine uptake thresholds for sensitivity analyses based on observed rollout efforts.^41^

| **Parameter Set** | **Older Adults** | **Younger Adults** |
| --- | --- | --- |
| Baseline | 0.9 | 0.7 |
| “Lower Uptake Targets” | 0.8 | 0.65 |
| “Extremely Low Uptake Targets” | 0.6 | 0.45 |

The “lower uptake targets” are based on the available current values averaged over the entire region. These do not necessarily reflect the maximum uptake levels achievable because many countries showed visible increasing trends.^41^ The “extremely low uptake targets” further adjusted down from the “lower uptake targets” – describing a condition where factors such as vaccine hesitancy may severely hinder uptake. We present the results of these sensitivity analyses over the next two pages. The results are broadly consistent with baseline analyses – with most countries’ optimal vaccine prioritisation strategies unchanged (i.e., staying in the diagonal axes, 87% for “lower uptake targets” and 61% for “extremely low uptake targets”, across decision-making metrics and roll-out scenarios). Using the “extremely low” uptake parameter set and under R3 and R4 – results are significantly noisier (58% changes in optimal changes in optimal vaccine allocation strategies across decision-making metrics and roll-out scenarios). Under R4 specifically, an advantage of V20 over V+ emerged. Countries facing low uptake issues may have different optimal vaccine prioritisation strategies than presented here in this study, but the overall advantage of V+ and V20 over V60 and V75 under R4 remain valid.

## **3.5 Vaccinating adolescents**

We did not include those younger than 20 years of age in any baseline analysis as most vaccine products currently available are not authorised for such age groups in most countries in the WHO European Region at the time of this study. As results from clinical trials conducted among adolescents emerge, some vaccines are now authorised for use in those between 12 and 15 years of age in a small number of countries.^42^ We thus expanded our analysis to include adolescents using the fastest vaccine roll-out scenario explored (i.e. R4) as it is the only one involving substantial vaccine surplus. We only expanded using V60 and V75 as the last groups vaccinated were younger adults, with whom it may make sense to potentially include adolescents.

# **References**

1 Davies N, Klepac P, Liu Y, Prem K, Jit M, Eggo R. Age-dependent effects in the transmission and control of COVID-19 epidemics. *Nature Medicine* 2020; **26**: 1205–11.

2 Levin AT, Hanage WP, Owusu-Boaitey N, Cochran KB, Walsh SP, Meyerowitz-Katz G. Assessing the age specificity of infection fatality rates for COVID-19: systematic review, meta-analysis, and public policy implications. *Eur J Epidemiol* 2020; **35**: 1123–38.

3 Prem K, Zandvoort K van, Klepac P, *et al.* Projecting contact matrices in 177 geographical regions: An update and comparison with empirical data for the COVID-19 era. *PLOS Computational Biology* 2021; **17**: e1009098.

4 United Nations Department of Economic and Social Affairs. 2019 Revision of World Population Prospects. https://population.un.org/wpp/ (accessed Nov 2, 2020).

5 Davies NG, Barnard RC, Jarvis CI, *et al.* Association of tiered restrictions and a second lockdown with COVID-19 deaths and hospital admissions in England: a modelling study. *The Lancet Infectious Diseases* 2021; **21**: 482–92.

6 Pearson CAB, Bozzani F, Procter SR, *et al.* Health impact and cost-effectiveness of COVID-19 vaccination in Sindh Province, Pakistan. *medRxiv* 2021; : 2021.02.24.21252338.

7 Davies N, Kucharski A, Eggo R, Gimma A, Edmunds W, COV C for the MM of ID. Effects of non-pharmaceutical interventions on COVID-19 cases, deaths, and demand for hospital services in the UK: a modelling study. *Lancet Public Health* 2020; **5**: e375–85.

8 Bi Q, Wu Y, Mei S, *et al.* Epidemiology and transmission of COVID-19 in 391 cases and 1286 of their close contacts in Shenzhen, China: a retrospective cohort study. *The Lancet Infectious Diseases* 2020; **20**: 911–9.

9 Liu Y, CMMID COVID-19 working group, Funk S, Flasche S. The contribution of pre-symptomatic infection to the transmission dynamics of COVID-2019. *Wellcome Open Research* 2020; **5**: 58.

10 Linton NM, Kobayashi T, Yang Y, *et al.* Incubation Period and Other Epidemiological Characteristics of 2019 Novel Coronavirus Infections with Right Truncation: A Statistical Analysis of Publicly Available Case Data. *Journal of Clinical Medicine* 2020; **9**: 538.

11 Nishiura H, Linton NM, Akhmetzhanov AR. Serial interval of novel coronavirus (COVID-19) infections. *International Journal of Infectious Diseases* 2020; **93**: 284–6.

12 Hall VJ, Foulkes S, Charlett A, *et al.* SARS-CoV-2 infection rates of antibody-positive compared with antibody-negative health-care workers in England: a large, multicentre, prospective cohort study (SIREN). *The Lancet* 2021; **397**: 1459–69.

13 Ritchie H, Mathieu E, Rodés-Guirao L, *et al.* Coronavirus Pandemic (COVID-19). *Our World in Data* 2020. https://ourworldindata.org/covid-deaths (accessed April 11, 2021).

14 Kyrgyzstan/Kazakhstan: New Rules for Tallying Covid-19 Data. Human Rights Watch. 2020; published online July 21. https://www.hrw.org/news/2020/07/21/kyrgyzstan/kazakhstan-new-rules-tallying-covid-19-data (accessed April 9, 2021).

15 Google Inc. COVID-19 Community Mobility Report. COVID-19 Community Mobility Report. 2020. https://www.google.com/covid19/mobility?hl=en (accessed May 18, 2020).

16 Hale T, Angrist N, Goldszmidt R, *et al.* A global panel database of pandemic policies (Oxford COVID-19 Government Response Tracker). *Nature Human Behaviour* 2021; : 1–10.

17 Gavi, the Vaccine Alliance. The COVAX Facility: Interim Distribution Forecast. 2021 https://www.gavi.org/sites/default/files/covid/covax/COVAX-Interim-Distribution-Forecast.pdf (accessed March 26, 2021).

18 World Health Organization. COVAX Announces additional deals to access promising COVID-19 vaccine candidates; plans global rollout starting Q1 2021. World Health Organization. 2020; published online Dec 18. https://www.who.int/news/item/18-12-2020-covax-announces-additional-deals-to-access-promising-covid-19-vaccine-candidates-plans-global-rollout-starting-q1-2021 (accessed April 11, 2021).

19 COVAX reaches over 100 economies, 42 days after first international delivery. World Health Organization. https://www.who.int/news/item/08-04-2021-covax-reaches-over-100-economies-42-days-after-first-international-delivery (accessed April 11, 2021).

20 Wouters OJ, Shadlen KC, Salcher-Konrad M, *et al.* Challenges in ensuring global access to COVID-19 vaccines: production, affordability, allocation, and deployment. *The Lancet* 2021; **397**: 1023–34.

21 Robinson E, Jones A, Lesser I, Daly M. International estimates of intended uptake and refusal of COVID-19 vaccines: A rapid systematic review and meta-analysis of large nationally representative samples. *Vaccine* 2021; **39**: 2024–34.

22 Department of Health and Social Care (UK). UK COVID-19 vaccine uptake plan. https://www.gov.uk/government/publications/covid-19-vaccination-uptake-plan/uk-covid-19-vaccine-uptake-plan (accessed April 18, 2021).

23 Oliver SE, Gargano JW, Marin M, *et al.* The Advisory Committee on Immunization Practices’ Interim Recommendation for Use of Pfizer-BioNTech COVID-19 Vaccine—United States, December 2020. *Morbidity and Mortality Weekly Report* 2020; **69**: 1922–4.

24 Pfizer-BioNTech. Vaccines and Related Biological Products Advisory Committee Meeting December 10, 2020. US Food and Drug Administration, 2020.

25 Education, Audiovisual and Culture Executive Agency. The organisation of school time in Europe: primary and general secondary education : 2019/20. LU: Publications Office, 2019 https://data.europa.eu/doi/10.2797/678694 (accessed April 26, 2021).

26 US CDC. Public Health Guidance for Community-Related Exposure. Centers for Disease Control and Prevention. 2020; published online Feb 11. https://www.cdc.gov/coronavirus/2019-ncov/php/public-health-recommendations.html (accessed April 26, 2021).

27 Eurostat. GISCO data distribution API - Countries 2020. https://gisco-services.ec.europa.eu/distribution/v1/countries-2020.html (accessed April 12, 2021).

28 Mishra S, Mindermann S, Sharma M, *et al.* Changing composition of SARS-CoV-2 lineages and rise of Delta variant in England. *EClinicalMedicine* 2021; **39**. DOI:10.1016/j.eclinm.2021.101064.

29 World Health Organization. Global Health Observatory data repository | Life tables by WHO region - Europe. World Health Organization. https://apps.who.int/gho/data/view.main.LIFEEUR?lang=en (accessed April 21, 2021).

30 Briggs AH, Goldstein DA, Kirwin E, *et al.* Estimating (quality-adjusted) life-year losses associated with deaths: With application to COVID-19. *Health Economics* 2021; **30**: 699–707.

31 Szende A, Janssen B, Cabases J, editors. Self-Reported Population Health: An International Perspective based on EQ-5D. Dordrecht: Springer Netherlands, 2014 DOI:10.1007/978-94-007-7596-1.

32 World Health Organization. WHO guide for standardization of economic evaluations of immunisation programmes. https://apps.who.int/iris/bitstream/handle/10665/329389/WHO-IVB-19.10-eng.pdf (accessed April 21, 2021).

33 Hoek AJ van, Underwood A, Jit M, Miller E, Edmunds WJ. The Impact of Pandemic Influenza H1N1 on Health-Related Quality of Life: A Prospective Population-Based Study. *PLOS ONE* 2011; **6**: e17030.

34 Halpin SJ, McIvor C, Whyatt G, *et al.* Postdischarge symptoms and rehabilitation needs in survivors of COVID-19 infection: A cross-sectional evaluation. *Journal of Medical Virology* 2021; **93**: 1013–22.

35 Bennett S, Tafuro J, Mayer J, *et al.* Clinical features and outcomes of adults with coronavirus disease 2019: A systematic review and pooled analysis of the literature. *International Journal of Clinical Practice* 2021; **75**: e13725.

36 Cuthbertson BH, Roughton S, Jenkinson D, MacLennan G, Vale L. Quality of life in the five years after intensive care: a cohort study. *Crit Care* 2010; **14**: R6.

37 Griffiths J, Hatch RA, Bishop J, *et al.* An exploration of social and economic outcome and associated health-related quality of life after critical illness in general intensive care unit survivors: a 12-month follow-up study. *Crit Care* 2013; **17**: R100.

38 Greenhalgh T, Knight M, A’Court C, Buxton M, Husain L. Management of post-acute covid-19 in primary care. *BMJ* 2020; **370**: m3026.

39 The World Bank. GDP per capita, PPP (current international $). https://data.worldbank.org/indicator/NY.GDP.PCAP.PP.CD (accessed April 21, 2021).

40 Wouters OJ, Shadlen KC, Salcher-Konrad M, *et al.* Challenges in ensuring global access to COVID-19 vaccines: production, affordability, allocation, and deployment. *The Lancet* 2021; **397**: 1023–34.

41 WHO/Europe Covid-19 vaccine programme monitor. https://worldhealthorg.shinyapps.io/EURO_COVID-19_vaccine_monitor/ (accessed Aug 18, 2021).

42 FDA Office of the Commissioner. FDA Authorizes Pfizer-BioNTech COVID-19 Vaccine for Emergency Use in Adolescents in Another Important Action in Fight Against Pandemic. FDA, 2021 https://www.fda.gov/news-events/press-announcements/coronavirus-covid-19-update-fda-authorizes-pfizer-biontech-covid-19-vaccine-emergency-use (accessed June 15, 2021).
